# A multifunctional human monoclonal neutralizing antibody that targets a unique conserved epitope on influenza HA

**DOI:** 10.1038/s41467-018-04704-9

**Published:** 2018-07-10

**Authors:** Sandhya Bangaru, Heng Zhang, Iuliia M. Gilchuk, Thomas G. Voss, Ryan P. Irving, Pavlo Gilchuk, Pranathi Matta, Xueyong Zhu, Shanshan Lang, Travis Nieusma, Juergen A. Richt, Randy A. Albrecht, Hillary A. Vanderven, Robin Bombardi, Stephen J. Kent, Andrew B. Ward, Ian A. Wilson, James E. Crowe

**Affiliations:** 1 Department of Pathology, Microbiology and Immunology, Vanderbilt University Medical Center, Nashville, TN 37232 USA; 2 Department of Integrative Structural and Computational Biology, The Scripps Research Institute, La Jolla, CA 92037 USA; 3 Beijing Synchrotron Radiation Facility, Institute of High Energy Physics, Chinese Academy of Sciences, Beijing, 100049 China; 4 The Vanderbilt Vaccine Center, Vanderbilt University Medical Center, Nashville, TN 37232 USA; 5 Department of Pediatrics, Vanderbilt University Medical Center, Nashville, TN 37232 USA; 6 College of Veterinary Medicine, Kansas State University, Manhattan, KS 66506 USA; 7 Department of Microbiology, Global Health and Emerging Pathogens Institute, at Icahn School of Medicine at Mount Sina, New York, NY 10029 USA; 8 Department of Microbiology and Immunology, Peter Doherty Institute for Infection and Immunity, University of Melbourne, Melbourne, VIC 3010 Australia; 9 The Skaggs Institute for Chemical Biology, The Scripps Research Institute, La Jolla, CA 92037 USA

## Abstract

The high rate of antigenic drift in seasonal influenza viruses necessitates frequent changes in vaccine composition. Recent seasonal H3 vaccines do not protect against swine-origin H3N2 variant (H3N2v) strains that recently have caused severe human infections. Here, we report a human *V*_*H*_*1-69* gene-encoded monoclonal antibody (mAb) designated H3v-47 that exhibits potent cross-reactive neutralization activity against human and swine H3N2 viruses that circulated since 1989. The crystal structure and electron microscopy reconstruction of H3v-47 Fab with the H3N2v hemagglutinin (HA) identify a unique epitope spanning the vestigial esterase and receptor-binding subdomains that is distinct from that of any known neutralizing antibody for influenza A H3 viruses. MAb H3v-47 functions largely by blocking viral egress from infected cells. Interestingly, H3v-47 also engages Fcγ receptor and mediates antibody dependent cellular cytotoxicity (ADCC). This newly identified conserved epitope can be used in design of novel immunogens for development of broadly protective H3 vaccines.

## Abstract (web summary)

Broadly neutralizing antibodies are potential therapeutics and can aid rational vaccine development. Here, the authors show that the human monoclonal antibody H3v-47 recognizes a highly conserved epitope in HA of H3N2 viruses, inhibits virus replication by blocking egress and other mechanisms, and protects mice from disease.

## Introduction

Influenza A H1 and H3 viruses are the two major viral influenza A subtypes that currently circulate in humans throughout the world. Since H3N2 viruses began circulating in the human population in 1968, they have caused higher morbidity and mortality rates during their dominant seasons than H1N1 or influenza B viruses, and therefore present a substantial health challenge^1–3^. Due to the high rate of antigenic drift and rapid evolution of human H3 viruses^4,5^, H3 vaccine strains need to be changed frequently to remain effective^6,7^. During the global 2014–2015 influenza season, H3N2 viruses predominated, accounting for more than 90% of all subtyped influenza A viruses^8,9^. In addition to seasonal infections, influenza A H3N2 variant (H3N2v) viruses of swine-origin have caused sporadic influenza infection in humans following direct exposure to swine^10^. These variant strains are triple reassortant viruses, with their genes originating from swine, avian, and human viruses, including the matrix (M) gene from the 2009 pandemic H1N1 virus^10^. In recent years, there has been a significant increase in the number of cases of H3N2v human infection, with more than 350 confirmed infections in the US since 2011^10,11^. H3N2v viruses have the capacity for efficient replication and transmission in ferrets^12^, and may pose a pandemic threat similar to that of the swine-origin 2009 pandemic H1N1 virus. More importantly, the H3N2v influenza viruses are antigenically distinct from seasonal influenza viruses^13^, and current vaccines are not effective in protecting against these variant strains^14–17^.

Influenza A viruses have two major surface glycoproteins, hemagglutinin (HA) and neuraminidase (NA). The trimeric HA protein is critical for facilitating virus entry and infection of host cells and is the major target of neutralizing antibodies^18,19^. The HA can be divided functionally into three major regions: (1) the receptor-binding subdomain (RBS), (2) the vestigial esterase subdomain located lower down on the HA globular head, and (3) the membrane-proximal stem region that is responsible for the low pH-triggered membrane fusion activity of HA in endosomal compartments^20,21^. Antigenic drift of the HA can act as a driving force for viruses to escape the human immune response^4,22^. HA antibodies can be grouped generally into those that recognize the head or stem domains. To inhibit viral infection, HA head-binding neutralizing antibodies usually block viral attachment, while HA-stem binding antibodies can prevent fusion between the viral and endosomal membranes and proteolytic activation of the HA0 precursor protein to HA1/HA2^23^. The classic antigenic sites on the immunodominant head domain of HA can be clustered generally in five sites in H3 HA designated A, B, C, D, or E^24,25^ or five sites in H1 HA designated Sa, Sb, Ca1, Ca2, or Cb^26^. Sites A and B (or Sa and Sb) are proximal to the receptor-binding site, sites C and D (Ca1 and Ca2) are at the subunit interface, and site E (Cb) is within the vestigial esterase domain. More recently, a broad influenza B antibody CR8071 that binds the vestigial esterase domain on HA head was shown to function primarily by inhibiting viral egress, similar to the function exhibited by NA inhibitors^23,27^. Another study demonstrated that HA antibodies possess some level of egress inhibition activity, possibly mediated by Fc-mediated steric hindrance of NA active site^28^.

In addition to the neutralization mechanisms mentioned above, antibodies to influenza proteins are capable of mediating additional effector functions through their Fc region using antibody dependent cellular cytotoxicity (ADCC)^29,30^ or antibody-dependent phagocytosis (ADP)^31^. ADCC is mediated when the Fc region of the antibodies that are bound to viral antigen on infected cells interact with FcγRIIIa (CD16a) on the surface of NK cells, resulting in crosslinking of FcγRIIIa. Subsequent NK cell activation triggers killing of virus-infected cells by releasing perforins and granzymes along with secretion of antiviral cytokines. In recent years, ADCC elicited by influenza antibodies has emerged as a factor that may contribute to the protective immunity afforded by stem antibodies in vivo^32^. However, neutralizing antibodies to the head domain that block receptor-binding sites typically are not thought to possess ADCC activity^32,33^.

Due to the significant health threat posed by H3N2 strains, it would be beneficial to develop a vaccine that targets a conserved epitope on HA and induces breadth of response against both human and swine H3 strains. Broadly neutralizing antibodies to influenza virus, often encoded by the human *V*_*H*_*1-69* germline gene segment^27,34,35^, generally target the structurally conserved HA stem domain^27,34–38^.

Here, we report that a human *V*_*H*_*1-69* gene-encoded mAb designated H3v-47, selected after immunization with a subunit experimental vaccine candidate based on the novel reassortant swine-origin vaccine strain (A/Minnesota/11/2010), possesses broadly neutralizing activity against both human and swine H3N2 viruses. In vivo studies with mice that were treated prophylactically or therapeutically with H3v-47 showed protection against weight loss and death following lethal virus challenge. Structural studies revealed that H3v-47 recognizes a unique epitope spanning the RBS and vestigial esterase region that is conserved in recent H3 viruses. Interestingly, H3v-47 functions primarily by inhibiting viral egress and also effectively engages Fc receptor to elicit ADCC activity. These findings identify a novel conserved epitope on H3 HA that can aid in development of a broad H3N2 vaccine and provide insights into unique antiviral approaches exploited by a vestigial esterase domain-binding antibody.

## Results

### Broad activity against human and swine H3N2 strains

We previously reported the isolation of H3v-reactive human monoclonal antibodies (mAbs) from donors vaccinated with an experimental H3N2v vaccine containing the A/Minnesota/11/2010 strain^39,40^. To investigate the breadth of one of these mAbs (H3v-47) further, we tested for binding by ELISA against a panel of recombinant HA molecules expressed from the HA genes of seasonal H3N2 strains that circulated during 1968–2014. MAb H3v-47 showed strong binding to H3N2 strains that circulated after 1989 (EC_50_ from 19 to 169 ng/mL) (Table 1). We also compared relative binding of H3v-47 Fab or IgG to a panel of seasonal H3 HAs by bio-layer interferometry. Consistent with the ELISA data, H3v-47 IgG exhibited high affinity to HA from seasonal H3 strains that occurred between 1989 and 2011 (*K*_d_ < 1 pM) whereas H3v-47 Fab displayed moderate-high affinity of binding to HA from H3 strains that circulated after 1989 (*K*_d_ from 224 nM to <1 pM) (Supplementary Table 1 and Supplementary Figure 1). Thus, H3v-47 can recognize HA molecules representing the diversity present in a 25-year time period from 1989 to 2014, during which significant antigenic drift occurred in human seasonal H3N2 viruses. Although binding was not detected for H3v-47 Fab to the HA of influenza strains isolated before 1989, its IgG demonstrated moderate avidity (*K*_d_ from 69 to 130 nM) for binding to these strains (Supplementary Table 1 and Supplementary Figure 1). H3v-47 did not show any binding to HA proteins from the four other influenza A subtypes tested (H1, H2, H5, or H7) (Supplementary Table 1).

**Table 1:** H3v-47 IgG breadth of HA binding and virus neutralization for human or swine H3N2 viruses

Virus	Strain	Binding EC_50_ (ng/mL)	Neutralization IC_50_ (ng/mL)
H3N2v	A/Minnesota/11/2010	4	8
Human H3N2 strains	A/Hong Kong/1/1968	>	>
	A/Victoria/3/1975*	>	>
	A/Bangkok/1/1979*	NT	>
	A/Leningrad/360/1986*	NT	>
	A/Beijing/353/1989*	NT	158
	A/Shandong/9/1993*	NT	223
	A/Wuhan/359/1995*	NT	127
	A/Sydney/5/1997*	24	385
	A/Panama/2007/1999*	20	2217
	A/Fujian/411/2002*	27	578
	A/New York/55/2004*	22	210
	A/California/7/2004*	35	NT
	A/Hiroshima/52/2005*	25	NT
	A/Wisconsin/67/2005*	NT	2741
	A/Brisbane/10/2007*	NT	1632
	A/Perth/16/2009*	19	711
	A/Victoria/361/2011*	19	1170
	A/Texas/50/2012*	31	598
	A/Switzerland/9715293/2013*	169	718
	A/Hong Kong/4801/2014*	NT	167
Swine H3N2 strains (Cluster I–IV)	A/Swine/Texas/4199-2/98	NT	1220
	A/Swine/Colorado/23619/99	NT	200
	A/Swine/Oklahoma/18089/99	NT	340
	A/Ohio/13/2012	NT	670

NT not tested. *WHO H3N2 vaccine strain for indicated year. '>' symbol indicates values >10,000

The in vitro neutralization activity for this mAb was largely consistent with the binding data. H3v-47 showed strong neutralization against all tested H3 strains isolated after 1989 but had considerably lower neutralizing activity against H3 strains isolated before 1989 (Table 1). We also compared relative neutralizing activity exhibited by H3v-47 Fab, F(ab′)_2_, or IgG against H3N2v A/Minnesota/11/2010 virus. While the F(ab′)_2_ form of H3v-47 neutralized at a similar potency to the IgG, H3v-47 Fab displayed a greater than 190-fold reduction in the IC_50_ value in comparison to IgG (<1 pM) (Supplementary Figure 2). Thus, H3v-47 requires the bivalency of IgG to potently neutralize the virus.

Since most healthy adult donors were partially immune to H3 seasonal viruses prior to experimental vaccination with the H3N2v-based subunit vaccine candidate, we cannot be sure that this antibody arose as a component of the primary response to the H3N2v antigen from a naive B cell. However, when tested for neutralization against swine viruses representing each antigenic cluster circulating in swine, A/Swine/Texas/4199-2/98 (cluster I), A/Swine/Colorado/23619/99 (cluster II), A/Swine/Oklahoma/18089/99 (cluster III), or A/Ohio/13/2012 (cluster IV), it was of interest that H3v-47 efficiently neutralized all four swine H3N2 strains (Table 1), which has not been reported previously for human H3 antibodies^34,36,38,41^. These data suggest that H3v-47 possesses broad-spectrum neutralizing activity for viruses of the H3 subtype and can efficiently inhibit both human and swine H3N2 viruses.

### Lethal H3N2v A/Minnesota/11/2010 mouse challenge model

We next sought to determine if mAb H3v-47 could prevent or treat infection with the H3N2v A/Minnesota/11/2010 virus. There is no established robust small animal infection model for this virus, so first we developed a lethal respiratory challenge model. Two groups of DBA/2 J mice (*n* = 15 per group) were inoculated i.n. with ∼1.2 × 10^5^ (high dose), or ∼1.2 × 10^4^ (low dose) FFU of A/Minnesota/11/2010 × −203 virus stock and monitored for 7 days for weight change kinetics. The results showed a dose-dependent response to the virus, and the highest dose caused mortality (Supplementary Figure 3a and 3b; note this figure shows body weight only for the animals that survived based on actual death between watches or endpoint for euthanasia). We also measured lung virus titers on dpi 1, 3, 5, 7, or 12 lungs (*n* = 3 per time point for each group), which showed the peak lung titers occurred on dpi 5, and mortality occurred during dpi 4–7 (Supplementary Figure 3c and 3d). We chose to use an intermediate dose in the prophylactic and therapeutic studies (6 × 10^4^ FFU) that was expected uniformly to cause ≥20% weight loss, a point beyond the mice could not recover, and to collect lungs for virus titer on dpi 4.

### Prophylactic and therapeutic administration of H3v-47 in mice

**Fig. 1:**
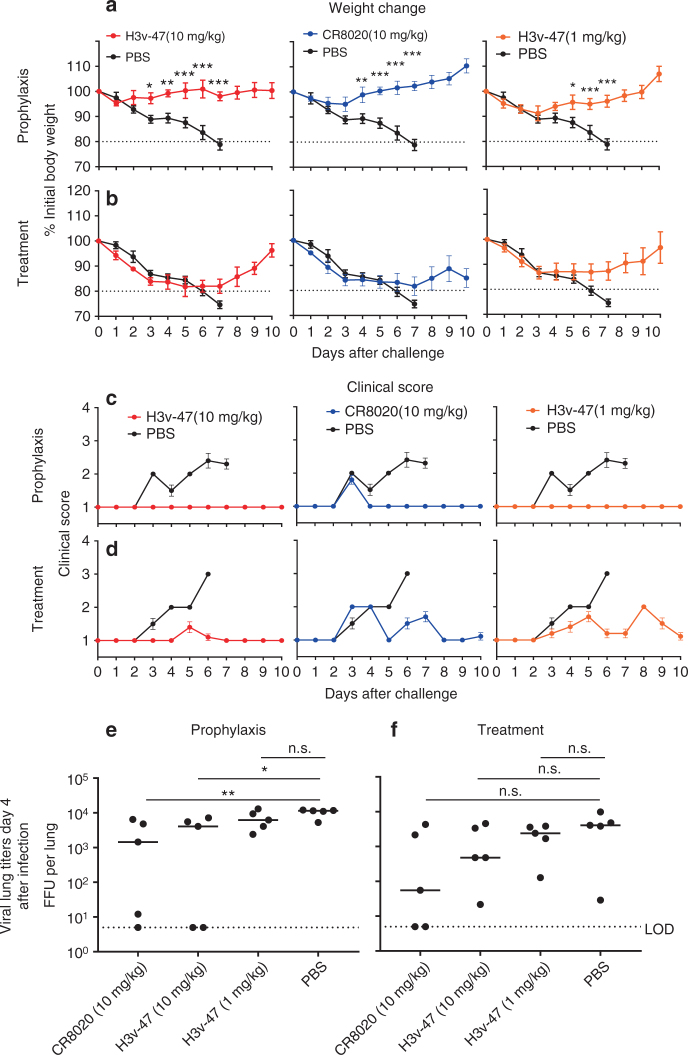
Prophylactic and therapeutic efficacy of mAb H3v-47 in mice. Groups of DBA/2J mice were inoculated i.p. with 10 (high dose) or 1 mg/kg (low dose) of mAb H3v-47 on the day before (day −1, prophylaxis), or on day one (day +1, treatment) after lethal respiratory (i.n. route) challenge with ∼ 6 × 10^4^ focus forming units (FFU) of A/Minnesota/11/2010 × −203 virus and monitored for 10 days. The control groups included mice treated with PBS (mock control) or the previously described mAb CR8020. The protective efficacy of mAbs was assessed by weight change kinetics (**a**, **b**), clinical score (**c**, **d**), and virus titers in lungs on 4 dpi (**e**, **f**). The dotted line (**a**, **b**) indicates the IACUC stipulated endpoint for humane euthanasia. Data in **a**, **b** shows body weight only for the animals that survived based on actual death between watches or endpoint for euthanasia, and represents the mean value ± SEM, using 10 mice per group. Each group was compared to the PBS-treated group using two-way ANOVA with Dunnett‘s post-hoc test for **a**, **b**, and one-way ANOVA with Dunnett‘s post-hoc test for **e**, **f**. Dots in **e**, **f** indicate individual mice (*n* = 5 mice per group). The median titer is shown with the line, and the dotted line indicates the limit of detection (LOD). *p < 0.05; **p < 0.01; ***p < 0.001 were considered significant

Groups of mice were inoculated i.p. with 10 (high dose) or 1 mg/kg (low dose) of mAb H3v-47 on the day before (day −1, prophylaxis), or on day one (day +1, treatment) after lethal respiratory (i.n. route) challenge and monitored for 10 days. The control groups included mice treated with PBS (mock control) or the previously described mAb CR8020. In the prophylaxis setting, the high dose of mAb H3v-47 completely protected against weight loss (Fig. 1a) and clinical disease (Fig. 1c). The low dose prophylaxis reduced weight loss and completely protected against clinical disease (Fig. 1a). In the treatment setting, the high and low dose of mAb H3v-47 protected against severe weight loss and a fatal endpoint (Fig. 1b), and both regimens had a moderate clinical score benefit comparable to that of CR8020 (Fig. 1d). MAb H3v-47 mediated a moderate but significant effect on virus titers in lungs on 4 dpi after prophylaxis after high or low dose administration (Fig. 1e), but neither mAb H3v-47 nor CR8020 had a significant effect on lung virus in the therapeutic setting (Fig. 1f). In summary, H3v-47-mediated protective clinical benefits in both prophylactic and therapeutic settings.

### Epitope map and structure of the H3v-47-HA complex

**Fig. 2:**
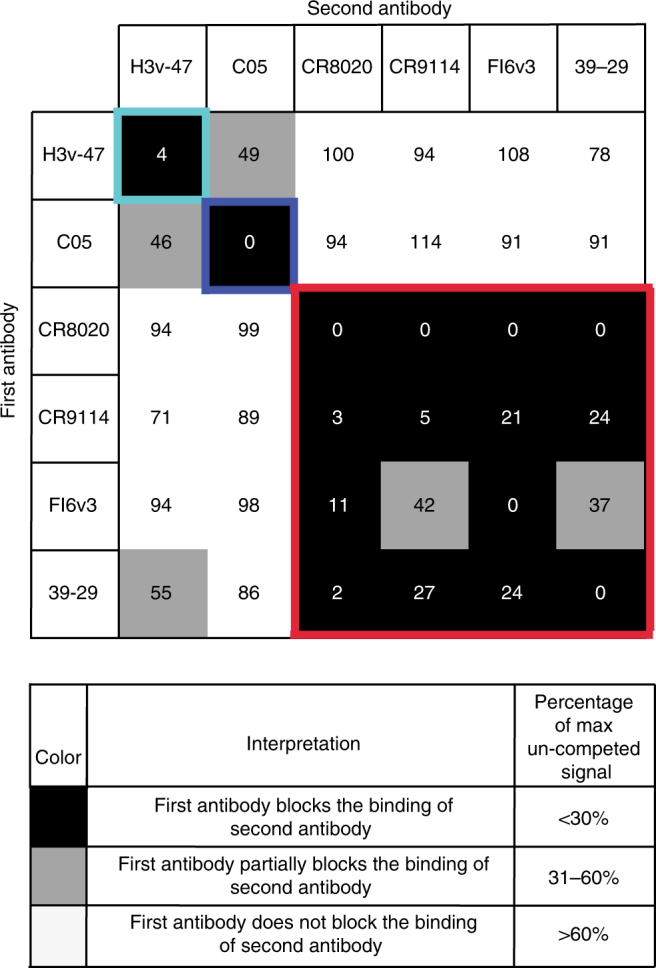
Competition binding of H3v-47 with other influenza head- or stem-binding antibodies. Competition-binding assays were performed using biolayer interferometry. The His-tagged A/Minnesota/11/2010 H3N2v HA was loaded onto Ni-NTA tips, and binding of two successively applied antibodies (IgG) was tested. MAb H3v-47 was competed against mAb C05, a receptor binding site mAb or each of four stem-binding antibodies: CR8020, CR9114, 39.29, or FI6v3. Numbers indicate normalized percent level of association to HA, compared to uncompeted control (100%). The colored boxes indicate each competition-binding group. There was partial overlap between group 1 and 2, shown in cyan and blue, respectively. Competition was not detected between H3v-47 and the stem antibodies indicated in the red competition-binding group box. The experiment was conducted twice independently

In our original report on isolation of H3v-reactive antibodies, it was apparent that H3v-47 does not bind to the RBS on HA head in a conventional manner due to its lack of hemagglutination inhibition (HAI) activity^39^. To examine if the antibody binds to the stem region, we used bio-layer interferometry to compete H3v-47 for binding against other known broad stem-binding antibodies including CR8020, CR9114, FI6v3, and 39.29 as well as the control RBS-binding antibody, C05. Surprisingly, we observed partial competition between H3v-47 and the RBS mAb C05, but did not detect competition with the stem-binding antibodies, indicating that H3v-47 does not bind to the HA stem region (Fig. 2).

**Fig. 3:**
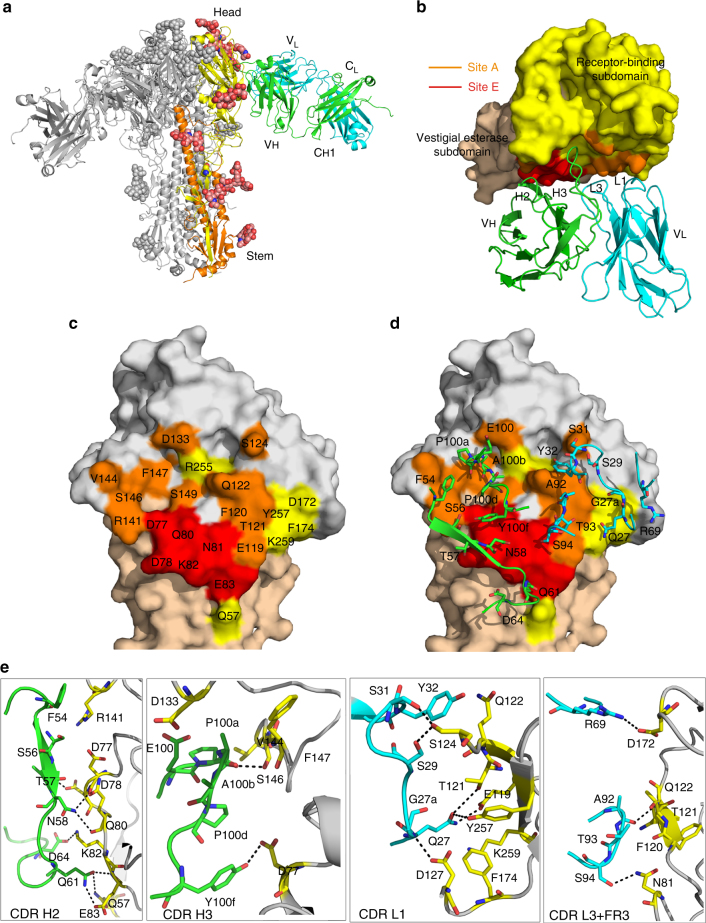
Crystal structure of antibody H3v-47 Fab in complex with H3N2v HA (A/Minnesota/11/2010) and identification of the epitope. **a** Overall structure of the H3v-47 Fab- H3N2v HA complex. One HA/Fab protomer of the trimeric complex is colored with HA1 in yellow, HA2 in orange, Fab heavy chain in green, and Fab light chain in cyan. N-linked glycans are depicted as colored balls representing their atom types (carbon in pink, oxygen in red and nitrogen in blue). The other two protomers are in gray, but the third Fab molecule is hidden behind the HA trimer. **b** Zoomed-in view of the interaction between H3N2v HA and H3v-47 Fab with color coding as in **a**. H3v-47 Fab binds to the region spanning the receptor-binding and vestigial esterase sub-domains (shown as a solid surface in yellow and wheat, respectively) mainly using CDRs H2, H3, L1, and L3. The contact regions ascribed to antigenic sites A and E are highlighted in orange or red, respectively. **c** H3v-47 epitope mapped onto the H3N2v HA surface. The footprint of antibody H3v-47 on the HA shows the central role of antigenic E site residues for H3v-47 binding. The interacting surface contributed by residues from antigenic sites A or E is colored in red or orange, respectively, whereas residues that contribute to the epitope outside of these two antigenic sites, are colored in yellow. **d** Antibody contact residues (sticks) that interact with the HA with CDRs H2, H3, L1, L3, and FR3 in the light chain. **e** Specific interactions (H-bonds, salt bridges, hydrophobic interactions, and VDW contacts) between H3v-47 and its footprint residues shown as sticks. The H-bonds and salt bridges are labeled using dash lines

To further investigate the antibody epitope, we determined the crystal structure of H3v-47 Fab in complex with H3N2 A/Minnesota/11/2010 (Minn2010/H3v) HA, at 3.57 Å resolution (Supplementary Table 2). Initial phases were obtained by molecular replacement using high-resolution structures that we also determined for H3v-47 Fab at 2.57 Å resolution and Minn2010/H3v HA-LSTc at 2.90 Å resolution (instead of the *apo* HA form at 3.15 Å, see below) (Supplementary Table 2). Despite the moderate resolution, the main chain and most side chains for the Fab and HA had interpretable electron density (Supplementary Figure 4). One HA protomer with one Fab was present in the asymmetric unit. The Fab binds to the globular head of HA below the RBS, in a region near the vestigial esterase subdomain (Fig. 3a, b and Supplementary Figure 4). Negative-stain EM reconstructions of the complex at 25.8 Å also showed that H3v-47 binds the HA head domain (Supplementary Figure 5). The position of the binding region suggested that H3v-47 cannot block the sialic acid-binding pocket, which is consistent with the observation that H3v-47 does not exhibit HAI activity. A total area of 1061 Å^2^ is buried at the antibody-antigen interface (511 Å^2^ on HA and 550 Å^2^ on the Fab), where the heavy and light chains contribute to 61% and 39% of the Fab buried surface area, respectively. Major conformational changes were not observed in the Minn2010/H3v HA upon complex formation when compared to the *apo* form of the HA, except for differences in a few side-chain rotamers.

H3v-47 targets a unique conformational epitope spanning the RBS and vestigial esterase region in the HA head mainly using the complementarity determining regions (CDRs) H2 and H3 from the heavy chain and L1 and L3 from the light chain (Fig. 3b). A total of 23 residues from Minn2010/H3v HA participate in intermolecular contacts (Fig. 3c), making a total of 18 hydrogen bonds (H bonds)/salt bridges with both heavy and light chains, as well as 96 van der Waals (VDW) interactions (Fig. 3d, e). PheH54 from CDR H2 (a signature residue on CDR H2 of *V*_*H*_*1-69*–encoded germline antibodies), and ProH100a, ProH100d, and TyrH100f from CDR H3, mediate the hydrophobic interactions with the HA. Footprint residues 77–83 in the vestigial esterase subdomain are classified as being part of the H3N2 antigenic site E, while residues 119–124, 133, 141–149 in the RBS are within antigenic site A (Fig. 3c). These two regions are bound by H3v-47 mainly through its heavy chain (CDRs H2 and H3) and light chain (CDRs L1, and L3), respectively, accounting for ~90% of the total contacts. For example, Lys82 in the antigenic site E makes one salt bridge by its side chain (Nζ) with AspH64 (Oε2), and eight VDW interactions with Gln61H and AspH64, mainly from its main chain (Fig. 3e). Moreover, the key residue Asp133 in the receptor binding pocket can make VDW contacts with GluH100 (Fig. 3e). In addition, ArgL69 side chain (Nη2) in framework region 3 (FR3) makes one salt bridge and one VDW contact with the side chain and main chain of Asp172 on the edge of the epitope, which may further stabilize the binding interface between the HA and Fab.

### Sequence analysis of natural variation in the H3v-47 epitope

The distinct neutralization profile of H3v-47 against H3N2 strains isolated before or after ~1989 indicates that naturally occurring variation in the epitope has a considerable effect on H3v-47 binding. The H3v-47 epitope is quite variable across human H3 virus strains in the National Center for Biotechnology Information (NCBI) Influenza database, with only 10 out of 23 contacting residues (77, 80, 119, 120, 141, 147, 149, 255, 257, and 259) being highly conserved (99–100%), and the remaining residues moderately conserved (7–82%) (Supplementary Table 3). The H3v-47 epitope is markedly less conserved than HA stem epitopes recognized by broadly neutralizing stem-specific antibodies, such as CR6261 and CR8020^33,34^. However, H3v-47 can tolerate natural variation in H3 vaccine strains spanning from 1989 to 2015. Variability within the footprint of H3 HAs may account for the large difference in H3v-47 binding affinities to H3 strains isolated before or after 1989. We performed a sequence alignment of 12 H3N2 HAs covering the 1968 pandemic to 2012 strains (Supplementary Table 4) that were used for binding assays (Supplementary Table 1). A lysine at position 82 in antigenic site E emerged in 1989 among the eight representative H3N2 strains that can be efficiently neutralized by H3v-47, whereas glutamic acid was present in H3N2 strains isolated before 1989. At position 124 in antigenic site A, serine and glycine are prevalent before and after 1989, respectively, except for aspartic acid in the Beijing 1989 and Shandong 1993 strains that have moderate Fab binding to H3v-47, but strong IgG avidity.

### Mutagenesis studies of the H3v-47 epitope

The two natural variants K82 and S124 in Minn2010/H3 HA were mutated to glutamic acid and glycine, respectively, to study their interactions with H3v-47. Binding of H3v-47 Fab was not detected either for the single K82E or double K82E/S124G mutant, while binding of S124G was much weaker (~50% of the wild-type protein, due to a faster off rate; Supplementary Figure 1 and Supplementary Figure 6a). However, both K82E and K82E/S124G retained as strong binding to H3v-47 IgG as the wild-type, which may suggest a bivalent mode of binding and the presence of avidity effects that compensate for weaker Fab binding, as observed for other antibodies to the HA head^42,43^. Meanwhile, most footprint residues in H3 subtype viruses are distinct from the residues at the same position in other virus subtypes (Supplementary Table 4), revealing why H3v-47 does not show cross-neutralization against other virus subtypes. Substitutions K82E and S124G likely would abolish the H-bonds (mediated by their side chains) and many VDW contacts (mainly by their main chains) with H3v-47 in the structure (Fig. 3e). K82E also changes the electrostatics on the HA surface, from positive Lys to negative Glu, causing unfavorable binding to H3v-47 (Supplementary Figs. 6c, d).

We attempted to isolate antibody resistant (“escape”) mutant viruses in laboratories at two different institutions. At neither site were we able to obtain escape mutants for mAb H3v-47, suggesting that viral escape to this antibody does not occur readily in vitro.

### N-glycosylation of H3v-47 combining site in Minn2010/H3v HA

Increase of N-glycosylation sites in the HA head domain is associated with shielding of the antigenic sites against antibody recognition during their evolution^44,45^. The 1968 pandemic H3 HA carries only two N-glycosylation sites on the globular head region (at positions 81 and 165), whereas seasonal human H3 HAs gradually have acquired up to six additional sites (at positions 63, 122, 126, 133, 144, and 165) during the period leading up to 2012. In Minn2010 H3v/HA, there are only four N-glycosylation sites in the globular head region (at positions 63, 126, 165, and 246; Supplementary Figure 7). The only two glycosylation sites N63 and N126 in the HA around the near the epitope do not make any contacts in the antibody-antigen interface (Supplementary Figure 6e).

Importantly, three H3v-47 epitope residues on Minn2010/H3v HA, Q122, D133, and V144, correspond to the acquired N-glycosylation site residues N122, N133, and N144 in recent seasonal H3 viruses (Supplementary Figures 6f and 7). Structural superposition of the present complex with the *apo*-structure of H3 HA from the seasonal A/Victoria/361/2011 (H3N2) virus (PDB code 4O5N) shows that the glycans at N122, N133, and N144 could make direct contacts with H3v-47, or could result in steric hindrance [n.b. the glycans in N122 and N144 are not modeled in this structure due to lack of electron density^43^, (Supplementary Figure 6f)]. However, it was reported that the binding of antibody FI6v3 can render an orientation change of the N38 glycan in HA from A/Aichi/2/1968 (H3N2) (PDB code 3ZTJ) compared to the HA *apo-*structure^34^. Similar glycan motions for formation of the complex with antibody CR9114 also have been suggested in N38 in H3 HA and N322 in influenza B HA^23,27^. Following this idea, the orientation of N-glycans attached to these epitope residues 122, 133, and 144 also might be variable and adaptable for H3v-47 binding in seasonal H3N2 viruses. Indeed, single mutation individually and even their combination of Q122, D133, or V144 of Minn2010/H3v HA to asparagine did not affect H3v-47 binding significantly as compared to the wild-type HA (Supplementary Figures 1 and 6a). Therefore, H3v-47 can overcome antigenic site masking by glycosylation at residues 122, 133, or 144, and therefore has broad cross-protective potential for seasonal H3N2 strains.

### Mechanism of neutralization by mAb H3v-47

Although mAb H3v-47 binds to the HA head region, it does not neutralize by blocking the RBS like most other head domain reactive neutralizing antibodies. To further explore the mechanism of neutralization mediated by H3v-47 recognition of this unusual epitope, trypsin digestion of Minn2010/H3v HA was performed in which the HA protein was exposed to a low pH (pH 5.0) to trigger the pH-induced conformational changes and acquire sensitivity to trypsin cleavage (Supplementary Figure 8). As expected, the low-pH-treated HA could be completely digested by trypsin in the absence of H3v-47 Fab. Binding of H3v-47 Fab to the HA did not protect the HA from degradation by trypsin when treated at pH 5.0, suggesting binding of H3v-47 does not prevent the low-pH induced conformational changes in HA as do stem-binding antibodies^23^.

**Fig. 4:**
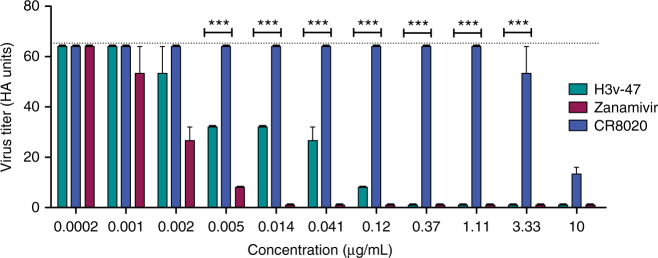
Inhibition of egress of A/Minnesota/11/2010 H3N2v virus by IgG of mAbs H3v-47 or CR8020 or by the small molecule neuraminidase inhibitor zanamivir. Twenty-hours prior to the experiment, MDCK cells were seeded on 6-well plates in DMEM + 5% FBS. The cells were washed twice with PBS and inoculated with a previously optimized amount of A/Minnesota/11/2010 H3N2v virus that is required to achieve 90–100% infection. After 1 h incubation at 37 °C, the cells were washed twice with PBS and replenished with 1.5 mL plain Opti-MEM I medium. After 3 h, the cells were washed again and replenished with 1.5 mL of Opti-MEM I medium containing serial dilutions of the antibodies or zanamivir. The cells were incubated at 37 °C for 12 h, and the supernatants were collected. The supernatants were diluted serially 11 times and added to an equal volume of 0.5% turkey RBCs in v-bottom plates to determine the virus titer by hemagglutination assay. Dotted line represents virus titer in supernatant in the absence of antibody treatment. The experiment was conducted three times independently. The hemagglutination assay to determine virus titer was also conducted three times independently (*n* = 3). The significance in the reduction of HA titer between H3v-47 and CR8020 was calculated at each concentration using 2-way ANOVA and displayed on the graph as ***(*P* < 0.001). Error bars indicate standard error of the mean (SEM)

We next tested whether H3v-47 functions through an alternative mechanism by blocking virus budding. We performed virus egress inhibition assays using MDCK cells inoculated with Minn2010/H3v virus that were treated with H3v-47 IgG at 3 h post inoculation to allow for unhindered virus attachment and fusion. The neuraminidase inhibitor zanamivir or the stem-specific neutralizing mAb CR8020 IgG were used in parallel as positive or negative control reagents, respectively, for egress inhibition. The assay was performed in the absence of trypsin to restrict viral replication to a single cycle. Infected cell supernatants were collected after 12 h, and the virus titers were determined by hemagglutination assay as HA units. A 50% reduction in the HA titer of virus compared to the untreated control was observed at concentrations of 4–10 ng/mL of the H3v-47 antibody (Fig. 4). This concentration correlates well with the IC_50_ of 8 ng/mL seen for H3v-47 against the Minn2010/H3v virus, suggesting that the main mechanism of neutralization by H3v-47 is inhibition of viral egress. As expected, zanamivir that blocks NA activity also showed potent inhibition of viral egress at similar molar concentrations as H3v-47, whereas the stem-binding antibody CR8020 did not mediate significant reduction in HA titer at concentrations as high as 3.3 µg/mL (Fig. 4). We did observe, however, some activity for CR8020 at the highest concentration tested (10 µg/mL). The cause for this activity is uncertain. This reduction in HA titer at higher antibody concentrations could be a result of minor inhibition of NA enzymatic activity exhibited by HA stem antibodies^28^.

**Fig. 5:**
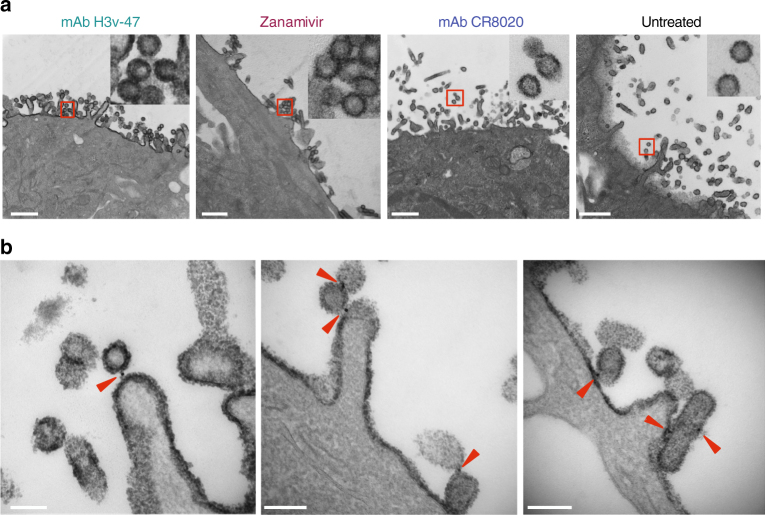
H3v-47 localizes to interfaces between virus and cell surface or between viral particles. **a** TEM images of the surface of MDCK cells inoculated with A/Minnesota/11/2010 H3N2v virus and infected cells were incubated with mAb H3v-47, zanamivir, mAb CR8020, or plain Opti-MEM at 3 h postinoculation and fixed for imaging at 14 h. For each image, the virus particles in the red squares are shown at higher magnification, below. Representative images of two independent experiments are shown. The white scale in each image represents 500 nm. **b** TEM images are shown of the surface of MDCK cells that were inoculated with virus and subsequently incubated with H3v-47 mAb similar to **a**, with addition of anti-human IgG conjugated to 10 nm gold particles at 13 h post-inoculation. The black opaque dots indicated by the red triangles represent the gold particles. The white scale in each image represents 100 nm. The experiment was conducted twice independently

To confirm that H3v-47 was acting at the step of virus egress from infected cells, we performed transmission electron microscopy (TEM) of MDCK cells inoculated with Minn2010/H3v virus and either untreated or treated with H3v-47 IgG, CR8020 IgG, or zanamivir at 3 h postinoculation. As expected, the major phenotype observed in zanamivir-treated cells was cell surface aggregation of fully formed virus particles, in contrast to the prevalence of released virus particles in the untreated cells (Fig. 5a and Supplementary Figure 9). H3v-47 mAb-treated cells also revealed virus aggregations on the cell surface, similar to those in the zanamivir-treated samples (Fig. 5a and Supplementary Figure 9a). In contrast, cells treated with stem mAb CR8020 showed individually budded particles resembling the pattern in untreated virus-infected cells (Fig. 5a and Supplementary Figure 9a). CR8020-treated cells also had considerably more virus particles inside the cells compared to zanamivir- or H3v-47-treated cells (Supplementary Figure 9a). Zanamivir-treated cells displayed more filamentous influenza particles on the cell surface compared to H3v-47-treated cells (Supplementary Figure 9a). Taken together, these data indicate that H3v-47 mAb functions by preventing viral egress in a manner comparable to zanamivir.

Antibodies to HA have been hypothesized to inhibit viral egress either by blocking NA enzymatic activity by Fc-mediated steric hindrance or by cross-linking newly formed virions or HA trimers on the cell surface^23^. To further elucidate the molecular mechanism by which H3v-47 inhibits egress, we performed TEM with gold labeling to determine the localization of H3v-47 during egress. The infected MDCK cell samples were prepared for TEM as before, but with the addition of anti-human IgG conjugated to gold particles 1 h before fixing cells. We observed that H3v-47 was predominantly present at the interface between the MDCK cells and newly budded virus particles or at virus–virus interfaces in comparison to CR8020 (Fig. 5b and Supplementary Figure 10). The ratio of the number of gold particles present at the interfaces (I) (virus–virus and surface–virus) to the total number of gold particles on the surface (S) was higher for H3v-47 (I/S = 5 of 8) compared to CR8020 (I/S = 2 of 8). Also, there was significantly less gold particle labeling of IgG on the cell surface of CR8020-treated cells (Supplementary Figure 10). Trace amounts of gold particles were present in zanamivir-treated and untreated cells (Supplementary Figure 10. Collectively, these results along with our previous observation that the F(ab′)_2_ form of H3v-47 had similar neutralization potency as the IgG (Supplementary Figure 2), suggests that H3v-47 may primarily function by tethering newly formed virions to the cell surface or to other viral particles rather than Fc-mediated steric hindrance of NA. However, it is possible that H3v-47 also possesses some level of neuraminidase enzymatic inhibition activity that contributes to its overall potency.

### H3v-47 mAb exhibits ADCC activity

**Fig. 6:**
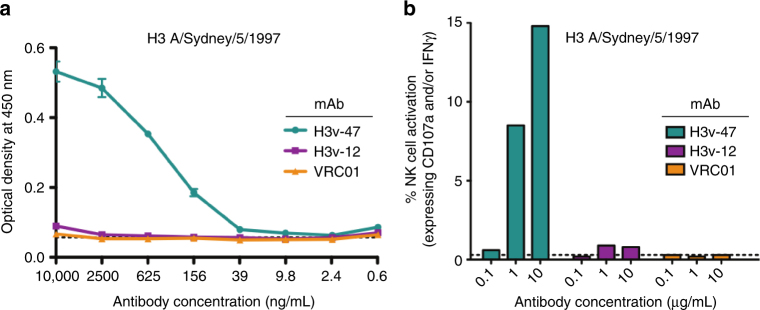
H3v-47 mAb exhibits ADCC activity. **a** Cross-linking of FcγRIIIa. Binding curves were obtained by performing ELISA with serial dilutions of each antibody (H3v-47, H3v-12, or control mAb VRC01 [to HIV]) onto HA-coated plates and assessing the ability of HA-bound mAbs to engage both Fc-binding sites on the soluble FcγRIIIa dimer. **b** Primary NK cell activation. Antibody (H3v-47, H3v-12, or VRC01) at 0.1, 1, or 10 µg/mL each were added independently on 96-well plates coated with purified A/Sydney/5/1997 H3 HA and incubated for 2 h. The plates were washed, and 5 × 10^5^ purified NK cells were added to each well. The cells were stained with anti-human CD107a allophycocyanin-H7 Ab, anti-human CD3 PerCP, anti-human CD56 allophycocyanin, and anti-IFNγ AF700. The data for 20,000–50,000 events were acquired using an LSRFortessa flow cytometer. The percentage of NK cell activation was calculated as the percentage of NK cells that expressed CD107a and/or IFNγ. The dotted lines in both **a** and **b** indicate the limit of detection. The results are representative of three independent experiments. Error bars indicate standard error of the mean (SEM)

HA stem-reactive antibodies frequently possess ADCC activity and depend on Fc receptor engagement to confer protection in vivo^32,46^. Recent work suggested that, while stem-binding antibodies potently elicit ADCC activity, the receptor-binding site antibodies seem to lack the ability to induce ADCC^46,47^. To examine if H3v-47, which targets the vestigial esterase subdomain on the HA head, could mediate ADCC activity, we first performed an ELISA using recombinant soluble (rs), dimeric, low-affinity ectodomains (rsFcγR) of FcγRIIIa as described^48^. These dimers require simultaneous engagement of two Fc domains to achieve stable binding detected by ELISA. We tested the ability of three mAbs [H3v-47, H3v-12 (HA-reactive), or VRC01 (an HIV-reactive control mAb)] bound to A/Sydney/5/1997 HA to simultaneous engage both binding sites on rsFcγR. Only H3v-47 bound to FcγR in this assay (Fig. 6a), suggesting its potential to mediate ADCC activity. Next, we examined the ability of these antibodies to mediate functional ADCC activity as measured by activation of primary CD3^−^ CD56^+^ NK cells following incubation with HA from A/Sydney/5/1997. NK cell activation was measured by intracellular IFN-γ expression and degranulation (CD107a expression). A concentration-dependent increase in the percentage of NK cell activation was observed for H3v-47 (14.8% NK cell activation at 10 µg/mL), while activation was not observed for H3v-12 or VRC01 at the highest concentration tested (10 µg/mL) (Fig. 6b). Collectively, these data show that mAb H3v-47 engages FcγRIIIa and induces ADCC activity, even though it binds to the head domain.

## Discussion

Recent research on influenza vaccinology has focused on the idea of identifying components of a broadly protective or universal vaccine that would protect better against seasonal drift virus strains or newly emerging pandemic strains. The most obvious conserved antigenic site for inclusion in such a vaccine studied to date is the stem region, which possesses highly conserved residues that can be recognized by human antibodies. Here, we describe the unique human mAb H3v-47 that mediates very broad neutralization of H3 influenza viruses by binding to an unusual conserved epitope on the side the H3 HA head domain. This antibody mediates inhibition of virus replication using multiple molecular mechanisms including inhibition of virus egress and mediation of ADCC activity. H3v-47 IgG protected animals against weight loss and disease when administered prophylactically or therapeutically. The detailed structural and functional data presented here suggest that engineered HA head domain antigens that focus the antibody response on the H3v-47 epitope could be an important component of a broadly protective H3 vaccine.

**Fig. 7:**
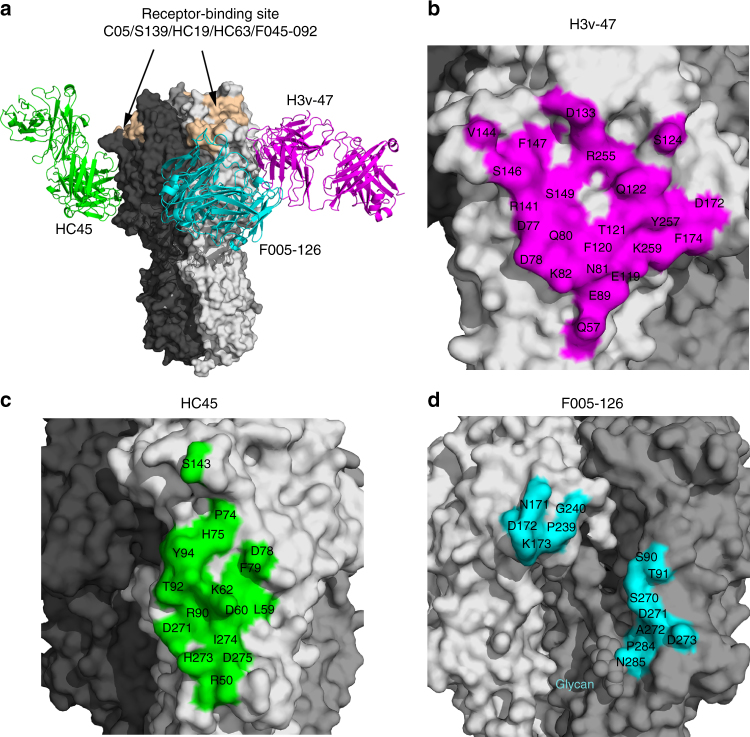
H3v-47 binds a unique epitope in the HA head domain. **a** Comparison of the binding site of H3v-47 (in magenta) wih structurally characterized H3-binding antibodies binding to the HA head. The three protomers of the HA trimer are shown as surface in light, middle or dark gray, respectively. The HAs from all these HA-antibody complexes are aligned. These antibodies include HC45 (PDB code 1QFU, green) and F005-126 (PDB code 3WHE, purple) binding to the globular head below the receptor-binding site (RBS). For comparison, mAbs C05 (PDB code 4FP8), S139 (PDB code 4GMS), HC19 (PDB code 2VIR), HC63 (PDB code 1KEN), and F045-092 (PDB code 4O58) all bind to the RBS. Because the epitopes of the RBS-binding antibodies overlap extensively, their epitopes (in wheat) are depicted approximately around the RBS, to indicate their relative location compared to H3v-47. **b**–**d** Comparison of the detailed epitopes recognized by H3v-47 (**b**), HC45 (**c**), and F005-126 (**d**)

Most neutralizing antibodies that bind to the HA head of H3N2 viruses recognize epitopes in or surrounding the RBS, mainly using interactions mediated by the antibody heavy chain (for instance, mAbs C05, F045-092, S139/1, HC19, and HC63)^49^. The binding sites of these antibodies overlap extensively (Fig. 7a), although they use very different angles of approach and relative orientations of their light and heavy chains. Three structurally characterized H3-neutralizing antibodies, HC45, BH151, and F005-126, bind to a region more distant from the RBS, using both light and heavy chains (Fig. 7a). The footprint of H3v-47 on HA is unique compared with these known antibodies, although their footprints slightly overlap (HA1 D78 in HC45, and D172 and K173 in F005-126) (Fig. 7b–d). Moreover, H3v-47 targets the H3 HA globular head with a distinct angle of approach from those of HC45 and F005-126 (Supplementary Figure 11). Although HC45 and BH151 also target the vestigial esterase subdomain, both are A/Aichi/2/1968 strain-specific H3 antibodies with narrow breadth of neutralization activity^50,51^. The footprint of F005-126 spans a cleft formed by two HA monomers in the head domain (Fig. 7d) and neutralizes certain H3 viruses isolated during the period from 1968 to 2004. On the other hand, H3v-47 spans the RBS and vestigial esterase subdomain, recognizing an epitope distinct from other H3-neutralizing antibodies.

**Fig. 8:**
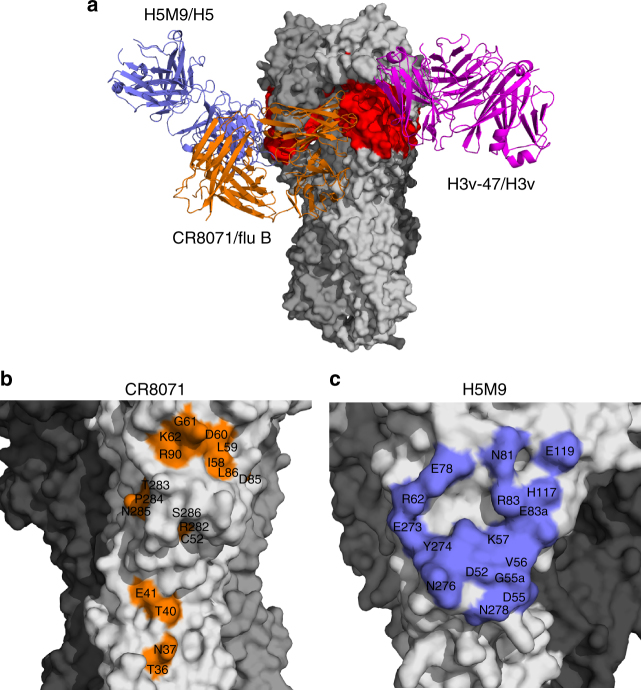
Vestigial esterase subdomain-binding antibodies mediate neutralization by diverse mechanisms. **a** Comparison of H3v-47 (magenta) with structurally characterized esterase subdomain-binding antibodies, including H5M9 binding to H5 HA (PDB code 4MHH, blue) and CR8071 (PDB code 4FQJ, orange) binding to influenza B HA. The HAs from all these complexes are aligned, and only the three protomers of A/Minnesota/11/2010 H3N2v HA trimer are shown as surface in light, middle or dark gray, respectively. The HAs from all these HA-antibody complexes are aligned. The vestigial esterase subdomain is highlighted in red. These antibodies target the HA vestigial esterase subdomain using different angles of approach. **b**-**c** The epitopes of CR8071 (**b**) and H5M9 (**c**) are mapped onto the surfaces of the corresponding HAs

Several antibodies were previously reported to bind to the vestigial esterase subdomain: the H3-neutralizing mAbs HC45 and BH151, the H5-neutralizing mAb H5M9, and the influenza type B neutralizing mAb CR8071^27,50–52^. H3v-47 targets the HA with a different approach angle from these antibodies (Fig. 8a). HC45 neutralizes viral infectivity by blocking receptor binding, as it has HAI activity^51^ and H5M9 prevents the low-pH induced conformational changes of the HA required for membrane fusion^52^. H3v-47 targets the vestigial esterase subdomain, but binds closer to the RBS than H5M9, and their epitopes only partly overlap (Fig. 8a, c). However, H3v-47 functions in a distinct manner, because it does not prevent the low-pH-induced conformational changes in the HA, nor does it have detectable HAI activity. Instead, H3v-47 functions primarily by interfering with progeny release similarly to the action of neuraminidase inhibitors like zanamivir. Egress inhibition activity has been recorded previously for an influenza type B antibody CR8071 that binds to the HA head domain and is oriented perpendicular to the long axis of the HA^27^ (Fig. 8a), recognizing a complex epitope (Fig. 8b). The suggested inhibition mechanism for mAb CR8071 was by cross-linking newly formed virions to each other and to HA on the cell membrane. It appears that H3v-47 functions in a similar manner as CR8071 by tethering newly formed virions on the cell surface despite binding to a different epitope, the vestigial esterase subdomain^23^.

The H3v-47 antibody exhibits broad inhibitory activity for human seasonal and swine H3 viruses isolated after ~1989, but variability in the epitope does occur. E82 or K82 residues are highly conserved (~100% conservation) across human and swine-origin H3N2 strains isolated before or after 1989 (Supplementary Figure 6b and Supplementary Table 5). The occurrence of the natural antigenic drift mutation E82K is consistent with the cross-neutralization activities of H3v-47 for human and swine H3 viruses isolated after ~1989. Moreover, the K82E variant disrupts binding of H3v-47 Fab to the HA, indicating that the substitution may play a major role in H3v-47 recognition and specificity, but the antibody retains binding to such variants in the IgG form by avidity effects. Because the natural antigenic variant E82K is highly conserved, H3v-47 has the potential to neutralize most circulating human and swine H3N2 viruses with this substitution.

Protective antibody responses to influenza vaccines typically are evaluated using HAI assay that detect receptor-blocking antibodies and by microneutralization assays that also detect stem-binding antibodies and their fusion inhibiting properties. These assays focus on detecting neutralizing antibodies that function principally through the action of the antibody variable domains. Fc-mediated ADCC activity has emerged as an important additional mechanism for protection by influenza stem-specific antibodies^23,32,46,53^. H3v-47 that binds to the vestigial esterase subdomain on the HA head but does not block the RBS was able to potently activate primary NK cells. The prophylactic efficacy of H3v-47 seen in vivo may be due in part to its ability to engage FcγRIIIa. A recent report showed the influenza B virus-neutralizing antibody 46B8 targeting the vestigial esterase domain can mediate ADCC, as well as block HA-mediated membrane fusion^54^. Additionally, H5 neutralizing mAb 100F4 that binds to HA head but does not block receptor binding was also shown to require Fc-mediated interactions to provide in vivo protection^55^. Collectively, these results suggest that Fc–FcγR interactions are important not only for HA-stem mAbs but also for HA-head mAbs that target the vestigial esterase domain.

The human mAb H3v-47 showed broad neutralization of human and swine H3N2 viruses by targeting a unique conserved epitope that has not previously been characterized as immunogenic on H3 viruses. The structure of H3v-47 in complex with H3v HA revealed that a region close to previously defined antigenic site E, containing the natural variant E82K, is a key site of vulnerability. H3v-47 functions uniquely by inhibiting viral egress and also by facilitating ADCC activity by engaging FcγRIIIa on NK cells. This epitope may induce production of broadly neutralizing antibodies against human and swine H3 viruses and enable design of more broadly protective H3 vaccines.

## Methods

### Culture of influenza virus

The original seed stocks for all the viruses were obtained from varying sources as recorded previously^39^. All of the working stocks were obtained by virus inoculation of MDCK cell culture monolayers (American Type Culture Collection, CCL-34) in plain Dulbecco Modified Eagle Medium (Gibco DMEM, Invitrogen, 11965) with 2 µg/mL of TPCK-trypsin. High titered stocks of Minn2010/H3v prepared in eggs were kindly provided by Richard Webby, St. Jude Children’s Research Hospital, for use in mouse challenge studies.

### Production of recombinant soluble HA proteins

The design and expression of recombinant HA proteins for binding studies were described previously^39^. Sequences encoding the HA genes were synthesized as soluble trimeric constructs by replacing the transmembrane and cytoplasmic domain sequences with cDNAs encoding the GCN4 trimerization domain and a His-tag at the C-terminus. Synthesized genes were subcloned into the pcDNA3.1(+) mammalian expression vector (Thermo Fisher Scientific) and expressed in FreeStyle 293-F cells (Thermo Fisher Scientific). Recombinant soluble biotinylated HA proteins for the *K*_d_ measurements and soluble HA proteins for crystallization and electron microscopy (EM) studies were produced using a baculovirus expression system, as described previously^56^.

### Production of H3v-47 IgG

The H3v-47 human hybridoma cell line was generated previously from a B cell in the blood of a subject immunized experimentally with an H3N2v subunit vaccine and cloned as previously described^39^. The clones were grown in 75 cm^2^ flasks to 70% confluency in hybridoma growth medium (ClonaCell-HY medium E from STEMCELL Technologies, 03805). The cells were expanded equally to four 225 cm^2^ flasks for antibody expression in serum-free medium (GIBCO Hybridoma-SFM, Invitrogen, 12045084). The supernatant was collected after 3 weeks and purified by affinity chromatography using protein G columns (GE Life Sciences, Protein G HP Columns). Purified H3v-47 IgG from hybridoma cell expression was used for all EC_50_ and IC_50_ studies, egress assays, TEM, and ADCC assays.

### Half maximal effective concentration (EC_50_) and half maximal inhibitory concentration (IC_50_) analysis

ELISAs were performed to obtain EC_50_ values for binding using 384-well plates coated with the HA of interest at a 2 μg/mL concentration and incubated overnight at 4 °C. The plates were blocked with 5% non-fat dry milk, 2% goat serum, and 0.1% Tween-20 in PBS for 1 h. Three-fold dilutions of the mAb, starting from 10 μg/mL, were added to the wells, incubated for 1 h, followed by a 1 h incubation of a 1:4000 dilution of anti-human IgG alkaline phosphatase conjugate (Meridian Life Science, W99008A). The plates were washed three times between each step with PBS containing 0.1% Tween-20. Phosphatase substrate solution (1 mg/mL *p*-nitrophenol phosphate in 1M Tris aminomethane) was added to the plates, incubated for 1 h, and the optical density values were measured at 405 nm wavelength on a BioTek plate reader. Each dilution was performed in triplicate, and the EC_50_ values were calculated in Prism software (GraphPad) using non-linear regression analysis.

For microneutralization, 50 μL of two-fold serial dilutions of each antibody, starting at a 20 μg/mL concentration, was incubated with 50 μL of 100 TCID_50_ of the virus in viral growth medium (VGM) for 1 h at RT. VGM consisted of plain DMEM with 2 μg/mL of TPCK-trypsin and 50 μg/mL gentamicin. The MDCK cell monolayer cultures were washed twice with 100 μL PBS containing 0.1% Tween-20, and the virus-antibody mixture then was added to cells and incubated for 32 h at 37 °C. The cells were washed again and fixed with 100 μL of 80% methanol/20% PBS. The presence of influenza nucleoprotein in the fixed cells was determined by ELISA using a 1:8000 dilution of mouse anti-NP antibody (BEI Resources, NR 4282) as the primary antibody and a 1:4000 dilution of goat anti-mouse alkaline phosphate conjugate as the secondary antibody (Thermo Fisher Scientific, 31320). Each dilution was tested in duplicate and the half-maximal inhibitory concentration (IC_50_) was determined by non-linear regression analysis of log_10_ [inhibitor] vs. response function, using Prism software (GraphPad). An IC_50_ value of 2 μg/mL was used as the threshold to determine the presence of functional neutralization. The experiments for determining EC_50_ (*n* *=* 4) and IC_50_ (*n* *=* 3) were conducted twice independently. Plaque reduction assay was used to determine IC_50_ values for H3v-47 against swine H3N2 strains. MDCK cells were seeded onto the 12-well tissue culture plates the day before inoculation. On the next day, 50 pfu of each H3N2 virus in 100 μL was first incubated with two-fold serially diluted antibodies for 1 h at 37 °C. The mixture was then added onto MDCK cells after removing their culture medium and incubated for 1 h at 37 °C. Plaque formation was assessed after a 2–3 day incubation at 37 °C. The inhibition mediated by H3v-47 antibody against each H3N2 virus was calculated as the percentage of the plaque reduction, and IC_50_ values were determined as the concentration at which 50% of plaque reduction was observed.

### Expression of recombinant H3v-47 Fab

The heavy and light chain variable regions of H3v-47 were cloned into the vector phCMV containing the C_H_1 region of an IgG1 appended to myc- and His-tags, respectively. The Fab fragment was expressed by transient co-transfection of the expression vector containing heavy chain and light chain into FreeStyle 293-F cells. Recombinant Fab was purified from culture supernatant using a nickel column followed by size exclusion chromatography using a Superdex 75 column (GE Healthcare). Purified Fab was measured by optical absorbance at 280 nm, and purity and integrity were analyzed by reducing and nonreducing SDS-PAGE. The purified H3v-47 Fab was concentrated to ~10 mg/mL for crystallization and *K*_d_ determination.

### *K*_d_ determination

*K*_d_ values were determined by bio-layer interferometry using an Octet RED instrument (ForteBio, Inc.), as described previously^36^. Biotinylated HA proteins were loaded onto streptavidin-coated biosensors in 1× kinetics buffer (1× PBS, pH 7.4, 0.01% bovine serum albumin [BSA], and 0.002% Tween 20) for 600 s. For measurement of *k*_on_, association of H3v-47 IgG/Fab was measured for 180–600 s by exposing the sensors to four to six concentrations of Fab in 1× kinetics buffer. For measurement of *k*_off_, dissociation of H3v-47 IgG/Fab was measured for 180–600 s in 1× kinetics buffer. Experiments were performed at 30 °C. The ratio of *k*_off_ to *k*_on_ was used to calculate the *K*_d_. All binding traces and curves used for fitting are reported in Supplementary Figure 1.

### Competition-binding groups

Biolayer interferometry using an Octet Red instrument (ForteBio) was used to perform competition-binding assays. The HA was loaded onto ForteBio Ni-NTA tips at a concentration of 20 μg/mL, and binding to two successively applied mAbs at 50 μg/mL was tested. All of the dilutions were made in 1 × kinetic buffer (ForteBio, 18-5032). The individual binding signal for each mAb was obtained after 300 s of a single association step of the mAb on to HA. For competition analysis, if binding of the first antibody blocked the binding of the second antibody by reducing its actual binding signal by more than 70%, it was defined as a competitor. If binding of the first antibody did not block the binding of the second antibody by reducing its actual binding signal by less than 30%, it was defined as a noncompetitor. A signal reduction between 30 and 70% was defined as partial blocking.

### Antibody treatment of mice

All protocols were reviewed and approved by the Vanderbilt University Institutional Animal Care and Use Committee. Briefly, 7-week old female DBA/2J mice (purchased from Jackson Labs) were sorted randomly into groups of 15 animals. Groups included prophylaxis (with antibody delivered one day before virus inoculation, designated day −1) or treatment (with antibody delivered 1 day after virus inoculation, designated day +1) using mock treatment with phosphate buffered saline, mAb H3v-47 or a positve control antibody (CR-8020). Treatment doses for mAb H3v-47 were 10 or 1 mg/kg and for control antibody was 10 mg/kg. All treatments were administered by the intraperitoneal route.

### Virus challenge and determination of efficacy of mAbs against infection and disease

All mice in the mAb prophylaxis or treatment studies were challenged with ∼ 6 × 10^4^ FFU of A/Minnesota/11/2010 × −203 via intranasal administration in 50 µL of sterile PBS. Animals were observed daily for signs of illness and for body weight changes. On day +4 after virus inoculation, five (5) mice per group were collected and lungs were collected for lung virus load analysis. Lungs were homogenized (Biospec Products, USA) in 1.0 mL of sterile PBS, and serial dilutions of tissue homogenate were evaluated for viral load by a focus forming assay on MDCK monolayers.

### Protease susceptibility assay

Experiments to test for trypsin susceptibility of the HA were performed as previously described^36^. For A/Minnesota/11/2010 (H3N2) HA, each reaction mixture contained 2.5 µg of the HA or 2.5 µg of the HA and a two-fold molar excess of H3v-47 Fab (two Fabs per HA protomer). Reaction mixtures were incubated at 37 °C for 1 h at pH 5.0 and at 8.0. After incubation, the reaction mixture was neutralized to pH 8.4. Trypsin then was added to all samples except controls at a final ratio of 1:20 (wt/wt) of trypsin to HA, and reaction mixtures were incubated overnight at 22 °C. Samples then were analyzed by non-reducing SDS-PAGE.

### Egress inhibition assay

MDCK cells were seeded in plain Dulbecco Modified Eagle Medium (Gibco DMEM, Invitrogen, 11965) containing 10% FBS on six-well plates overnight. The cells were washed three times with PBS and 200 µL of previously optimized virus titer required to achieve 90–100% infection was added to the cells and incubated for 1 h at 37 °C degrees with periodic shaking. One hour after inoculation, the cells were washed once and replenished with 2 mL Opti-MEM I with GlutaMAX medium (Gibco, Life Technologies, 51985) per well and incubated for two more hours at 37 °C degrees. After a total of 3 h after inoculation, the cells were washed again and replenished with medium containing three-fold serial dilutions of H3v-47 antibody, CR8020 antibody or zanamivir (Relenza, NDC0713068101), starting at the highest concentration of 10 µg/mL. Trypsin was not added to the cells, in order to restrict the infection to a single cycle. The plates were incubated for 12 h at 37 °C degrees and the supernatants were collected for performing the HA assay. For HA assay, we used turkey red blood cells (Rockland Immunochemicals, R313) that were washed and diluted to 0.5% in 2.5% sodium citrate. A volume of 50 µL of the supernatants that were serially diluted two-fold in medium were incubated with 50 µL of the 0.5% turkey red blood cells in v-bottom plates for 1 h at 4 °C. The HA titers in the supernatants were calculated based on the lowest supernatant dilution at which hemagglutination was observed. The HA titers were performed in triplicate, and results shown are an average of three wells.

### Transmission electron microscopy

MDCK cells were inoculated with A/Minnesota/11/2010 H3N2v virus and subsequently incubated with H3v-47 IgG (5 µg/mL), CR8020 IgG (10 µg/mL), Zanamivir, or plain Opti-MEM I medium at 3 h postinoculation using the same conditions as the egress assay. Trypsin was not added to cells to preserve surface filaments, in order to assess egress inhibition. For gold labeling, the samples were treated identically except for the addition of goat-anti-human IgG conjugated to 10 nm gold particles (EMS, 25208) at 13 h postinoculation. At 14 h after virus inoculation, samples were washed with 0.1 M cacodylate buffer and fixed in 2.5% gluteraldehyde in 0.1 M cacodylate buffer, pH 7.4 at room temperature (RT) for 1 h then transferred to 4 °C, overnight. The samples were washed in 0.1 M cacodylate buffer, then incubated 1 h in 1% osmium tetraoxide at RT, then washed with 0.1 M cacodylate buffer. Subsequently, the samples were dehydrated through a graded ethanol series and then three exchanges of 100% ethanol. Next, the samples were incubated for 5 min in 100% ethanol and propylene oxide (PO) followed by two exchanges of pure PO. Samples then were infiltrated with 25% Epon 812 resin and 75% PO for 30 min at RT. Next, they were infiltrated with Epon 812 resin and PO [1:1] for 1 h at RT, and then overnight at RT. The next day, the samples underwent a 3:1 (resin: PO) exchange for 3–4 h, then were incubated with pure epoxy resin overnight. Samples then were incubated in two more changes of pure epoxy resin and allowed to polymerize at 60 °C for 48 h. For imaging, 70–80 nm ultra-thin sections were cut and collected on 300-mesh copper grids and post-stained with 2% uranyl acetate, and then with Reynold’s lead citrate. Samples subsequently were imaged on a Philips/FEI Tecnai T12 electron microscope at varying magnifications. Specimens were processed for TEM and imaged in the Vanderbilt Cell Imaging Shared Resource – Research Electron Microscopy facility.

### Dimeric recombinant soluble FcγRIIIa (CD16a) binding ELISA

A dimeric recombinant soluble FcγRIIIa (rsFcγRIIIa) ELISA was used to model the need for ADCC-inducing Abs to cross link FcγRIIIa^48^. A 96-well ELISA plate was coated with 50 ng of purified influenza HA protein from H3N2 A/Sydney/5/1997 (Sino Biological Inc., 40149-V08B) protein overnight at 4 °C in PBS. The plate was washed twice with PBST and blocked with 140 µL of PBS 1 mM EDTA, 1% BSA (PBSE/BSA) for 1 h at 37 °C. The plate then was washed twice with PBST and 50 µL of H3v-47 diluted in PBSE/BSA was added into duplicate wells, with concentrations ranging from 40 μg/mL to 2.4 ng/mL. Negative control wells with HA protein and PBS alone also were included (“no antibody control”). Following addition of H3v-47 or PBS, the plate was incubated at 37 °C for 1 h. The ELISA plate was washed five times with PBST and 50 µL of 0.1 µg/mL rsFcγRIIIa (V176) dimer was added to the wells, then the plate was incubated for 1 h at 37 °C. Pierce High Sensitivity Streptavidin-HRP (Thermo Fisher Scientific, 21130) was diluted 1:10,000 in PBSE/BSA, added to all wells, and the plate was incubated at 37 °C for 1 h. The plate was washed eight times with PBST and blotted dry. A volume of 50 µL of TMB substrate was added to each well and the plate was developed for 4–8 min in the dark. The reaction was stopped with 1 M HCl and the plate read at an absorbance of 450 nm.

### NK cell activation assay

A total of 96-well ELISA plates were coated with 600 ng of purified influenza H3N2 A/Sydney/5/1997 HA protein (Sino Biological Inc., 40149-V08B) overnight at 4 °C in PBS. The wells were washed five times with PBS and incubated with 10 μg/mL, 1 μg/mL or 0.1 μg/mL of H3v-47 diluted in PBS for 2 h at 37 °C. Negative control wells with HA protein and PBS alone also were included (“no antibody control”). Plates were washed seven times with PBS, and 5 × 10^5^ purified NK cells were added to each well. Healthy donor PBMCs were isolated with Ficoll-Paque PLUS (GE Healthcare Life Sciences, 171440). NK cells were purified from freshly isolated PBMCs using the EasySep human NK cell enrichment kit (STEMCELL Technologies, 19055) and resuspended in RF10 medium (RPMI 1640 supplemented with 10% FCS, penicillin, streptomycin, and l-glutamine). Mouse anti-human CD107a allophycocyanin-H7 antibody (clone H4A3, BD Biosciences, 561343; used at a 1:160 dilution), 5 μg/mL brefeldin A (Sigma-Aldrich, B6542), and 5 μg/mL monensin (BD GolgiStop; BD Biosciences, 554724) were added to the cells and incubated for 5 h at 37 °C in 5% CO_2_. Purified NK cells then were incubated with 1 mM EDTA to minimize cell adherence to the plates, anti-human CD3 PerCP (clone SP34-2, BD Biosciences, 552851, used at a 1:40 dilution), and anti-human CD56 allophycocyanin (clone B159, BD Biosciences, 555518; used at a 1:20 dilution) for 30 min at room temperature in the dark. Cells were fixed with 1% formaldehyde for 10 min and permeabilized with FACS permeabilizing solution two (BD Biosciences, 347692) for 10 min. PBMCs then were incubated at room temperature for 1 h with anti-human IFNγ AF700 (clone B27; BD Biosciences, 561024; used at a 1:400 dilution) in the dark. Finally, cells were again fixed with 1% formaldehyde and data sets for 20,000–50,000 events were acquired using an LSRFortessa flow cytometer (BD Biosciences). The experiment was performed twice independently, with duplicate wells tested in each experiment.

### Crystallization and X-ray structure determination

Apo H3v-47 Fab and apo Minn2010/H3v HA crystals were grown using our automated Rigaku Crystalmation robotic system at The Scripps Research Institute by sitting drop vapor diffusion. Crystals of H3v-47 Fab (10 mg/mL) grew at 20 °C using 20% (w/v) polyethylene glycol (PEG) 6000, 0.1 M sodium citrate (pH 5.0) as precipitant. Crystals were cryo-protected in mother liquor supplemented with 15% (w/v) glycerol, flash cooled, and stored in liquid nitrogen until data collection. Crystals of Minn2010/H3v HA (10 mg/ml) grew at 20 °C with 0.2 M calcium acetate, 18% (w/v) polyethylene glycol PEG 8000, 0.1 M sodium cacodylate (pH 6.5) as precipitant. Complexes of the HA with human receptor analog LSTc were obtained by soaking HA crystals in precipitant solution that contained glycan ligands in a final concentration of 5 mM. Crystals were cryo-protected in mother liquor supplemented with 15% (w/v) glycerol, flash cooled, and stored in liquid nitrogen until data collection. X-ray diffraction data for the H3v-47 Fab apo, Minn2010/H3v HA apo, and HA-LSTc complex were collected to 2.57, 3.15, and 2.90 Å resolutions at beamline 23ID-D at the Advanced Photon Source (APS), respectively. The diffraction data from H3v-47 Fab and Minn2010/H3v HA were processed using HKL2000 in spacegroups *P3*_*1*_ and *I2*_*1*_*3*, respectively^57^. The crystal structure of H3v-47 Fab was determined by molecular replacement with Phaser^58^ using the variable and constant domains of an Fab in the PDB (4Q9Q) as a search model; two Fabs were found in the asymmetric unit. The model was iteratively rebuilt using Coot^59^ and refined in Phenix^60^. Refinement parameters included rigid body refinement (for each Ig domain), simulated annealing, and restrained refinement including TLS refinement (for each Ig domain). The initial Minn2010/H3v HA apo structure was solved by molecular replacement method using Phaser^58^ with an H3 HA structure (PDB code 2YP2) as a search model. The H3v HA apo structure was used as the starting model for structure determination of the H3v HA-LSTc complex structure. Structure refinement was carried out in Phenix^60^ and model building with COOT^59^.

The H3v-47-Minn2010/H3v HA complex was prepared by adding recombinant H3v-47 Fab to HA in a 1.2:1 molar ratio and the saturated complex was purified by gel filtration. Crystals of the complex (~8 mg/mL) were grown by sitting drop vapor diffusion at 20 °C with 10% (v/v) 2-methyl-2,4-pentanediol (MPD), 0.1 M MES (pH 5.0) as precipitant. Crystals were cryo-protected in mother liquor supplemented with 10% MPD, flash cooled, and stored in liquid nitrogen until data collection. X-ray diffraction data for the H3v-47-Minn2010/H3v HA complex were collected to 3.57 Å resolution at APS beamline 23ID-D and processed in spacegroup *P2*_*1*_*3* using HKL2000^57^. The structure was determined by molecular replacement with Phaser^58^ using H3v-47 Fab *apo* and Minn2010/H3v HA-LSTc complex (instead of *apo* form) as the search model. The model was iteratively rebuilt using Coot^59^ and refined in Phenix^60^. Refinement parameters included rigid body refinement (for the HA and for the variable and constant domains of each Fab), restrained refinement including TLS refinement (for the HA and for the variable and constant domains of Fab), using the high-resolution HA and Fab as reference models. Final refinement statistics are summarized in Supplementary Table 2.

### Structural analyses

Hydrogen bonds and van der Waals contacts were calculated using HBPLUS^61^ and CONTACSYM^62^, respectively. The surface area buried upon Fab binding was calculated using MS^63^. MacPyMOL (DeLano Scientific) was used to render figures. Kabat numbering was applied to the coordinate files using the AbNum server^64^. The final coordinates were validated using the JCSG quality control server (v2.8), which includes MolProbity^65^.

### EM structures of H3v-47-HA complexes

Complexes were prepared by mixing the HA with saturating amounts of H3v-47 Fab and FI6v Fab at room temperature for 2 h. The complex was purified by size exclusion chromatography using a Superdex 200 10/300 GL column (GE Healthcare) to remove excess Fab. The complex was diluted to 2 ug/ml, applied to freshly glow discharged 400 mesh carbon coated copper grids, and negatively stained with 2% uranyl formate. The complexes were imaged at 52K times magnification on an FEI T12 at 120 kV TEM resulting in a pixel size of 2.05 A/pixel (calibrated using catalase crystal diffraction) with a dose of 24.7 e/Å^2^. All data were collected using Leginon Multi-Scale Imaging (MSI-raster 3.1) software^66^. The T12 TEM was equipped with a Teitz F416 4k × 4k CMOS. Images were collected while tilting the stage from 0 to 50 degrees in 5-degree increments to increase sampling of different angular orientations of the particles. DoGpicker^67^ was used to automatically select particles from 599 raw micrographs that were then binned by two resulting in 4.10 Å /pixel size and placed into 80 × 80 pixel boxes. Particles were aligned with Iterative MRA-MSA and ISAC1 resulting in a final stack of 23,175 raw particles. Class averages from ISAC were used to create a common lines initial model in EMAN2^68^. Model refinement was conducted in EMAN1^69^ resulting in a 25.8 Å resolution reconstruction based on a 0.5 FSC cutoff value.

### Sequence analysis of the antibody epitopes

The full-length and non-redundant influenza A HA sequences were downloaded from the Influenza Virus Resource at the National Center for Biotechnology Information (NCBI) database^70^. At the time of download (December 31, 2015), the dataset includes 4859 human H3 HA sequences. The sequences were aligned using MUSCLE^71^ with default parameters.

### Data availability

Atomic coordinates and structure factors for the crystal structures of H3v-47 Fab, A/Minnesota/11/2010 (H3N2) HA in *apo* form, the HA in complex with LSTc, and the HA in complex with H3v-47 Fab have been deposited in the Protein Data Bank with the accession codes 5XRQ, 5XRT, 5XRS, and 5W42, respectively. The authors declare that all other data supporting the findings of this study are available within the article and its Supplementary Information files, or are available from the authors upon request.

## Electronic supplementary material

Supplementary Information
