# Supplementary material for: A multifunctional human monoclonal neutralizing antibody that targets a unique conserved epitope on influenza HA
Source: Nat Commun. 2018 Jul 10;9:2669. doi: 10.1038/s41467-018-04704-9 (PMC6039445; doi:10.1038/s41467-018-04704-9)
Supplement: Supplementary file 1 — Supplementary Information [file 41467_2018_4704_MOESM1_ESM.pdf]

Supplementary Figures and Tables

Supplementary Table 1. Binding kinetics of H3v-47 IgG or Fab to diverse influenza HA subtypes

Subtype	Strain	Binding	
		Fab	IgG
H3N2v	A/Minnesota/11/2010	++	++++
Human H3N2	A/Hong Kong/1/1968	-	+
	A/Victoria/3/1975*	-	+
	A/Bangkok/1/1979*	-	+
	A/Leningrad/360/1986*	-	+
	A/Beijing/353/1989*	+	++++
	A/Shandong/9/1993*	+	++++
	A/Panama/2007/1999*	+++	++++
	A/Wyoming/3/2003	++++	++++
	A/Brisbane/10/2007*	+++	++++
	A/Perth/16/2009*	++++	++++
	A/Victoria/361/2011*	++++	++++
H1N1	A/Texas/36/1991	-	-
H2N2	A/Japan/305/1957	-	-
H5N1	A/Vietnam/1203/2004	-	-
H7N9	A/Shanghai/2/2013	-	-

*WHO H3N2 vaccine strain for indicated year.
Dissociation constants:
+ indicates K_d of 50–500 nM;
++ indicates K_d of 5–50 nM;
+++ indicates K_d of 0–5 nM;
++++ indicates K_d <1 pM
- indicates binding not detected at 500–4,000 nM Fab or IgG level.

Supplementary Table 2. X-ray data collection and refinement statistics

Data collection	H3v-47 Fab	Minn2010/H3v HA	H3v-47-Minn2010/H3v HA complex	Minn2010/H3v HA-LSTc complex
Beamline	APS 23ID-D	APS 23ID-D	APS 23ID-D	APS 23ID-D
Wavelength (Å)	1.033	1.033	1.033	1.033
Space group	$P3_1$	$I2_13$	$P2_13$	$I2_13$
Unit cell parameters (Å)	$a = b$ = 135.1, c =78.53	$a = b = c$ = 293.0	$a = b = c$ =194.6	$a = b = c$ = 293.0
Resolution (Å)	50.0 – 2.57 (2.61 – 2.57) [a]	50.0 – 3.15 (3.20 – 3.15) [a]	50.0 – 3.57 (3.63 – 3.57) [a]	50.0 – 2.90 (2.95 – 2.90) [a]
Observations	94,605	176,611	56,585	178,807
Unique reflections	50,148 (2,143) [a]	82,265 (4,534) [a]	29,479 (1,441) [a]	91,393 (4,740) [a]
R_{merge} (%) [b]	9.3 (41.2) [a]	17.5 (86.9) [a]	11.9 (78.4) [a]	16.4 (89.9) [a]
R_{pim} (%) [b]	5.8 (25.6) [a]	5.1 (67.7) [a]	4.5 (58.4) [a]	5.0 (78.7) [a]
I/σ	8.1 (1.0) [a]	22.4 (1.1) [a]	14.8 (1.0) [a]	23.6 (1.3) [a]
Completeness (%)	98.2 (83.4) [a]	100.0 (100.0) [a]	100.0 (100.0) [a]	100.0 (100.0) [a]
Redundancy	3.4 (2.7) [a]	20.1 (17.2) [a]	13.1 (10.1) [a]	19.5 (18.2) [a]
Z_a [c]	2	4	1	4
**Refinement statistics**				
Resolution	46.9 – 2.60	48.9-3.15	38.9-3.57	48.7-2.91
No. reflections	48,572	71,713	29,378	91,356
R_{cryst} (%) [d]	18.0	19.9	19.5	18.7
R_{free} (%) [e]	21.4	24.5	22.7	22.5
Protein atoms	6,533	15,658	7,260	15,620
Carbohydrate atoms	-	1,299	325	1,368
Waters	105	0	0	0
Average B-values ($Å^2$)				
Overall	19	100	140 (HA)/124 (Fab)	70
Wilson	42	87	132	78
RMSD from ideal geometry				
Bond length (Å)	0.010	0.005	0.016	0.006
Bond angles (°)	1.17	0.90	2.04	0.93
Ramachandran statistics (%) [f]				
Favored	95.4	94.2	94.8	95.2
Outliers	0.4	0.8	0.7	0.8
PDB ID	5XRQ	5XRT	5W42	5XRS

[a] Numbers in parenthesis refer to the highest resolution shell.

[b] $R_{merge} = \Sigma_{hkl} \Sigma_i | I_{hkl,i} - \langle I_{hkl} \rangle | / \Sigma_{hkl} \Sigma_i I_{hkl,I}$ and $R_{pim} = \Sigma_{hkl} [1/(n-1)]^{1/2} \Sigma_i | I_{hkl,i} - \langle I_{hkl} \rangle | / \Sigma_{hkl} \Sigma_i I_{hkl,I}$, where $I_{hkl,i}$ is the scaled intensity of the ith measurement of reflection h, k, l, $\langle I_{hkl} \rangle$ is the average intensity for that reflection, and n is the redundancy.

[c] Za is the number of molecules per asymmetric unit. Four HAs come from an NCS trimer and a protomer from a crystallographic trimer .

[d] $R_{cryst} = \Sigma | F_o - F_c | / \Sigma | F_o |$ x 100, where F_o and F_c are the observed and calculated structure factors, respectively. [e] R_{free} was calculated as for R_{cryst}, but on a random test set comprising 5% of the data excluded from refinement. [f] Calculated using MolProbity[1]

Supplementary Table 3. Conservation of H3v-47 footprint across human H3 HAs. All full-length and non-redundant human H3N2 HA sequences were downloaded from the Influenza Virus Resource at the NCBI database for sequence conservation analysis.

Residue HA1	H3 Consensus[a]	Minn2010/H3v Sequence[b]	H3 Conservation (%)[c]
57	Q	Q	60
77	D	D	99
78	G	D	7
80	Q	Q	99
81	N	N	80
82	K	K	72
83	K	E	29
119	E	E	99
120	F	F	100
121	N	T	18
122	N	Q	7
124	S	S	62
133	N	D	14
141	R	R	100
144	N	V	17
146	S	S	82
147	F	F	100
149	S	S	99
172	E	D	35
174	F	F	99
255	R	R	100
257	Y	Y	100
259	K	K	99

[a]Most common residue at position by simple majority across 4,859 available human H3 HA sequences (including 48 sequences from lab strains) at time of download (December 31, 2015).

[b]Residue at that position in Minn2010/H3v crystal structure.

[c]Percent conservation values of Minn2010/H3v sequences among all aligned human H3 HAs.

Supplementary Table 4. Minn2010/H3v HA contact residues with antibody H3v-47 and conservation in other influenza HA subtypes and strains. Residues in A/Minnesota/11/2010 H3N2v HA used in mutational studies (K82E, S124G, Q122N, D133N and V144N) are highlighted in red.

Subtype	Strain	Contact residues in HA epitope																						
		57	77	78	80	81	82	83	119	120	121	122	124	133	141	144	146	147	149	172	174	255	257	259
H3N2	A/Minnesota/11/2010	Q	D	D	Q	N	K	E	E	F	T	Q	S	D	R	V	S	F	S	D	F	R	Y	K
H3N2	A/Beijing/353/1989	R	-	G	-	-	-	-	-	-	I	N	D	S	-	K	-	-	-	G	-	-	-	-
H3N2	A/Shandong/9/1993	R	-	G	-	-	-	-	-	-	I	N	D	-	-	N	-	-	-	G	-	-	-	-
H3N2	A/Panama/2007/1999	-	-	G	-	-	-	-	-	-	N	N	-	N	-	N	-	-	-	E	-	-	-	-
H3N2	A/Wyoming/3/2003	-	-	G	-	-	-	K	-	-	N	N	-	N	-	N	-	-	-	E	-	-	-	-
H3N2	A/Brisbane/10/2007	-	-	G	-	-	-	K	-	-	N	N	-	N	-	N	-	-	-	E	-	-	-	-
H3N2	A/Perth/16/2009	-	-	G	-	-	-	K	-	-	N	N	-	N	-	K	-	-	-	E	-	-	-	-
H3N2	A/Victoria/361/2011	-	-	G	-	-	-	K	-	-	N	N	-	N	-	N	-	-	-	E	-	-	-	-
H3N2	A/Hong Kong/1/1968	R	-	V	-	-	E	T	-	-	I	T	G	N	-	G	G	-	-	-	-	-	-	-
H3N2	A/Victoria/3/1975	R	-	G	-	-	E	K	-	-	I	N	G	N	-	D	G	-	-	-	S	-	-	-
H3N2	A/Bangkok/1/1979	R	-	G	-	-	E	K	-	-	I	N	G	S	-	D	-	-	-	G	-	-	-	-
H3N2	A/Leningrad/360/1986	R	-	G	-	-	E	K	-	-	I	N	G	S	-	-	-	-	-	G	-	-	-	-
H1N1	A/Texas/36/1991	A	E	S	F	S	-	S	-	R	F	E	-	T	H	K	-	-	R	K	K	W	-	A
H2N2	A/Japan/305/1957	P	-	R	L	S	V	-	-	K	V	K	-	T	V	N	-	-	R	S	E	E		-
H5N1	A/Vietnam/1203/2004	K	-	E	I	-	V	-	-	K	I	-	-	S	Y	K	-	-	R	N	E	E	-	-
H7N9	A/Shanghai/2/2013	K	-	Q	L	E	F	S	D	K	E	A	-	N	-	S	-	-	A	R	S	D	S	F

"-": same residue at this position as in A/Minnesota/11/2010 H3N2v HA.

Supplementary Table 5. Comparison of H3v-47 footprint residues in A/Minnesota/11/2010 H3N2v HA with corresponding residues in representative swine H3 HAs covering the four clusters.

Subtype	Strain	HA1 contact residues in H3 HAs																						
		57	77	78	80	81	82	83	119	120	121	122	124	133	141	144	146	147	149	172	174	255	257	259
H3N2	A/Minnesota/11/2010	Q	D	D	Q	N	K	E	E	F	T	Q	S	D	R	V	S	F	S	D	F	R	Y	K
H3N2	A/Swine/Texas/4199-2/98	R	-	G	-	-	-	-	-	-	-	N	D	-	-	-	-	-	-	-	-	-	-	-
H3N2	A/Swine/Colorado/23619/99	-	-	G	-	-	-	-	-	-	N	N	-	-	-	N	-	-	-	E	-	-	-	-
H3N2	A/Swine/Oklahoma/18089/99	-	-	G	-	-	-	-	-	-	-	K	G	-	-	-	-	-	-	-	-	-	-	-
H3N2	A/Ohio/13/2012	-	-	-	-	-	-	-	-	-	-	-	-	-	-	-	-	-	-	-	-	-	-	-
H3N2	A/Swine/Colorado/1/77	R	-	G	-	-	E	K	-	-	I	N	G	N	-	D	G	-	-	-	S	-	-	-
H3N2	A/swine/Wisconsin/194/1980	R	-	G	-	-	E	K	-	-	F	N	G	N	-	D	-	-	-	-	-	-	-	-

"-" indicates the same residue at this position as in A/Minnesota/11/2010 H3N2v HA.

Supplementary Figure 1. Binding curves for reported K_d values for H3v-47 to different H3 HAs. Blue curves are the experimental traces obtained from bio-layer interferometry experiments, and red curves are the best global fits to the data used to calculate the K_d values. Binding curves for IgG and Fab were best fitted using the 1:2 (Bivalent Analyte) and 1:1 ligand binding model, respectively.

A/Minnesota/11/2010 (H3N2) binding to H3v-47 Fab, K_d = 44.3 nM

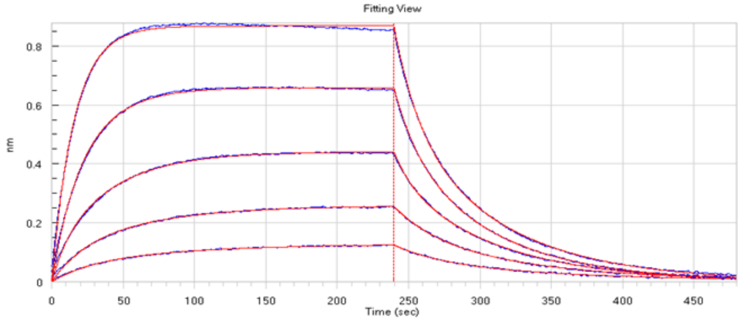

A/Minnesota/11/2010 (H3N2) binding to H3v-47 IgG, K_d < 1pM

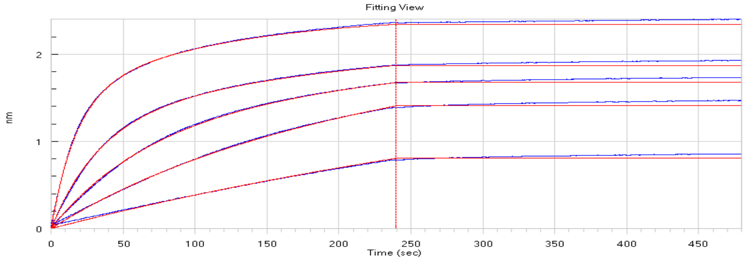

A/Minnesota/11/2010 (H3N2) K82E mutant binding to H3v-47 IgG, K_d < 1 pM

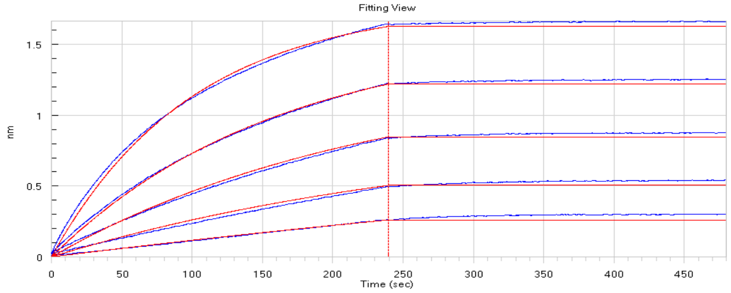

A/Minnesota/11/2010 (H3N2) S124G mutant binding to H3v-47 Fab, K_d = 94.0 nM

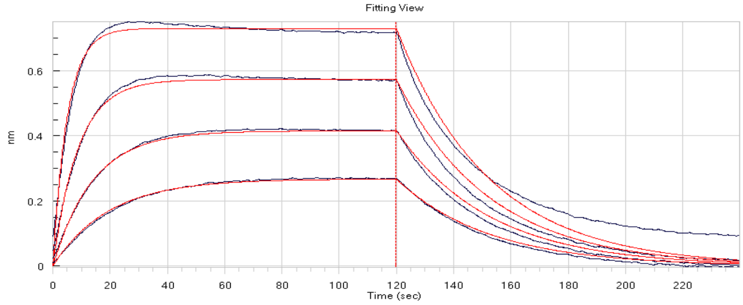

A/Minnesota/11/2010 (H3N2) S124G mutant binding to H3v-47 IgG, K_d < 1 pM

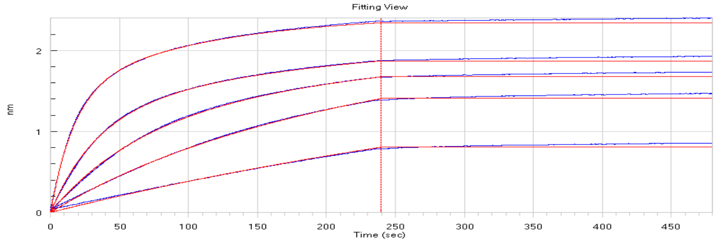

A/Minnesota/11/2010 (H3N2) Q122N mutant binding to H3v-47 Fab, K_d = 57.2 nM

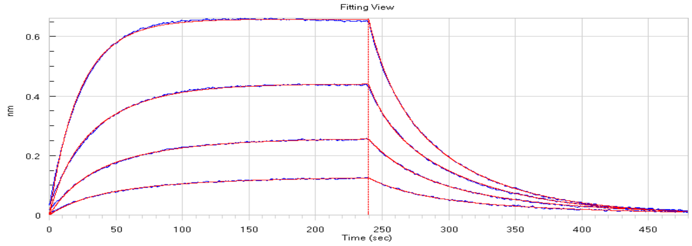

A/Minnesota/11/2010 (H3N2) Q122N mutant binding to H3v-47 IgG, K_d < 1 pM

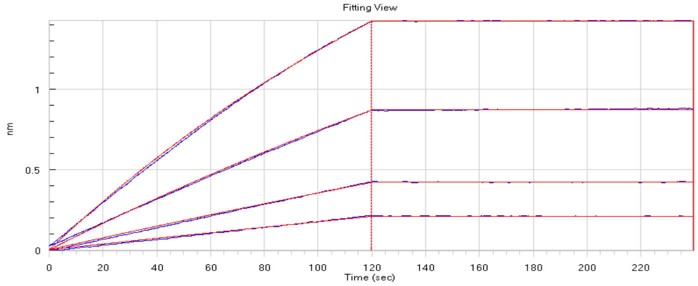

A/Minnesota/11/2010 (H3N2) D133N mutant binding to H3v-47 Fab, K_d = 37.9 nM

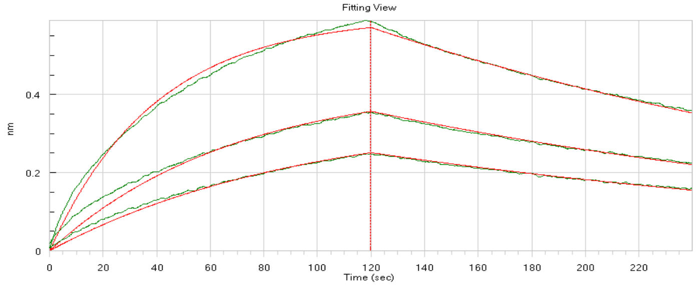

A/Minnesota/11/2010 (H3N2) D133N mutant binding to H3v-47 IgG, $K_d < 1$ pM

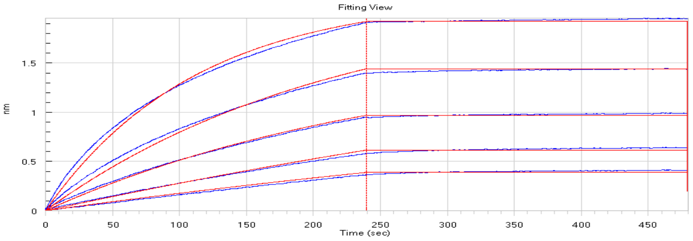

A/Minnesota/11/2010 (H3N2) V144N mutant binding to H3v-47 Fab, $K_d = 107.3$ nM

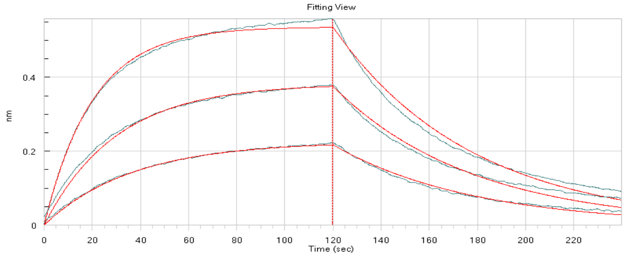

A/Minnesota/11/2010 (H3N2) V144N mutant binding to H3v-47 IgG, $K_d < 1$ pM

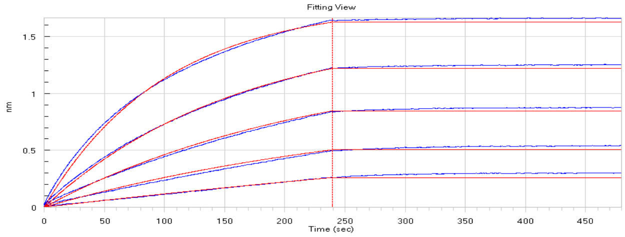

A/Minnesota/11/2010 (H3N2) Q122N/D133N/V144N mutant binding to H3v-47 Fab, $K_d = 207$ nM

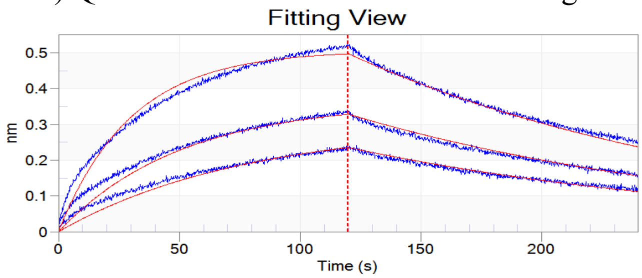

A/Minnesota/11/2010 (H3N2) Q122N/D133N/V144N mutant binding to H3v-47 IgG, $K_d < 1$ pM

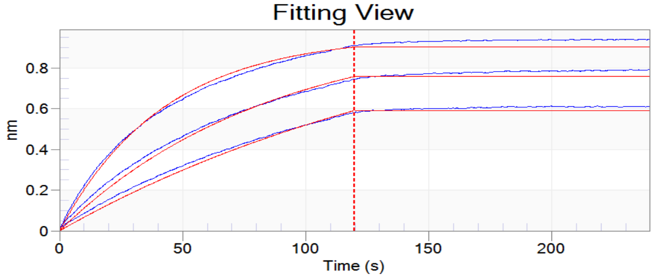

A/Beijing/353/1989 (H3N2) binding to H3v-47 Fab, K_d = 224 nM

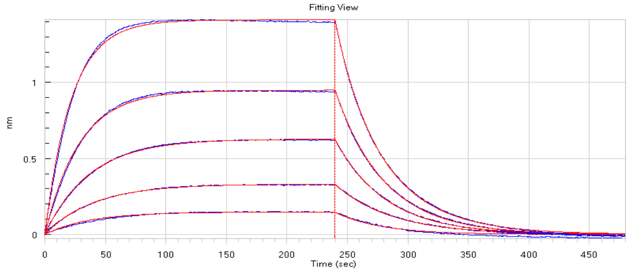

A/Beijing/353/1989 (H3N2) binding to H3v-47 IgG, K_d < 1 pM

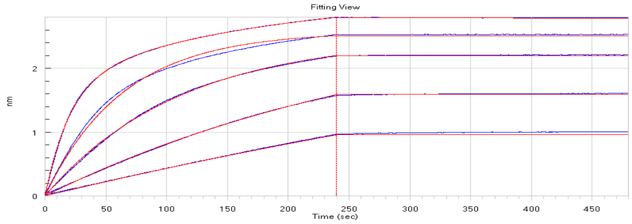

A/Shandong/9/1993 (H3N2) binding to H3v-47 Fab, K_d = 211 nM

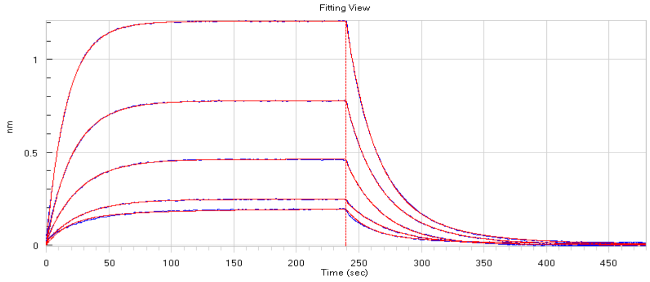

A/Shandong/9/1993 (H3N2) binding to H3v-47 IgG, K_d < 1pM

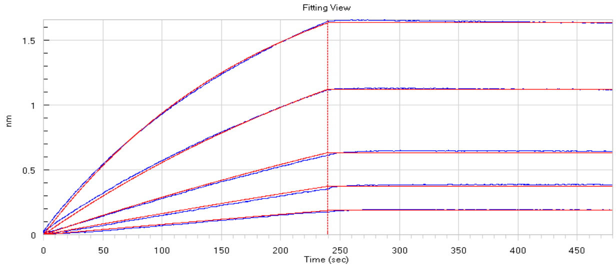

A/Wyoming/3/2003 (H3N2) binding to H3v-47 Fab, K_d< 1 pM

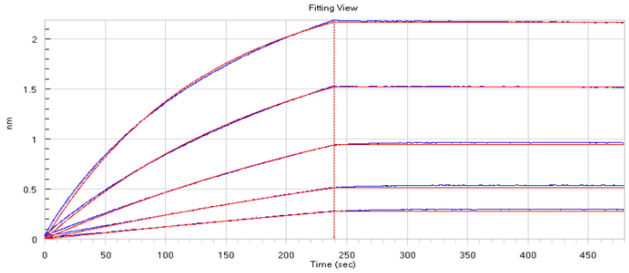

A/Wyoming/3/2003 (H3N2) binding to H3v-47 IgG, K_d < 1 pM

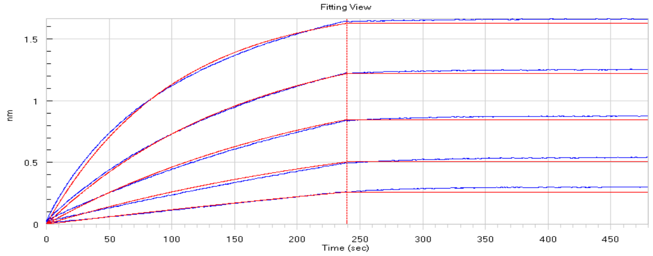

A/Brisbane/10/2007 (H3N2) binding to H3v-47 Fab, K_d = 1.97 nM

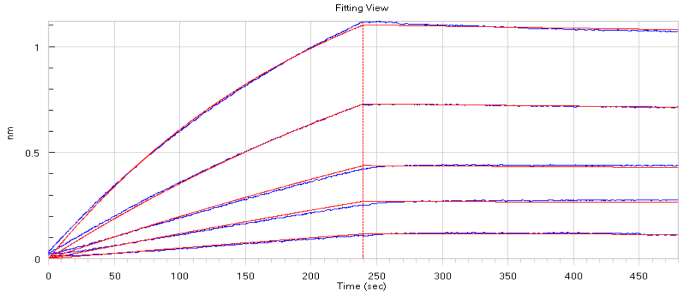

A/Brisbane/10/2007 (H3N2) binding to H3v-47 IgG, K_d < 1 pM

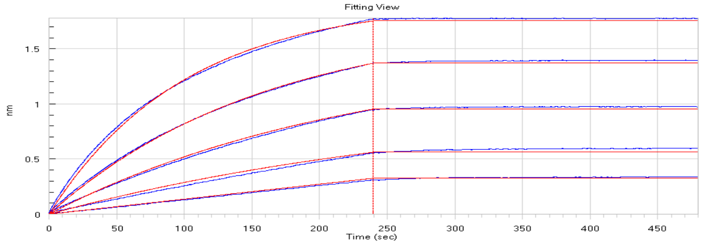

A/Panama/2007/1999 (H3N2) binding to H3v-47 Fab, K_d = 2.19 nM

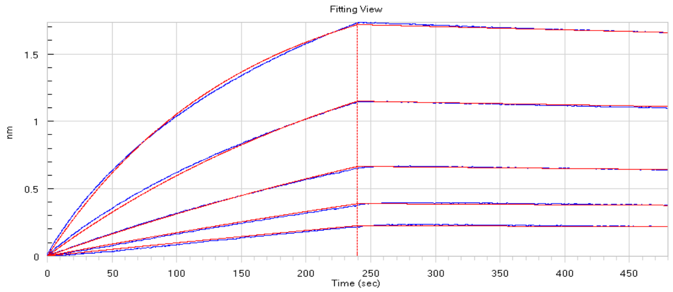

A/Panama/2007/1999 (H3N2) binding to H3v-47 IgG, K_d < 1 pM

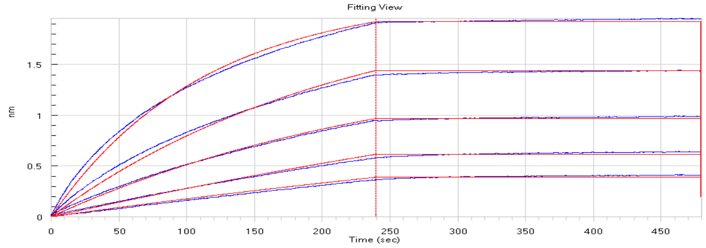

A/Victoria/361/2011 (H3N2) binding to H3v-47 Fab, K_d < 1 pM

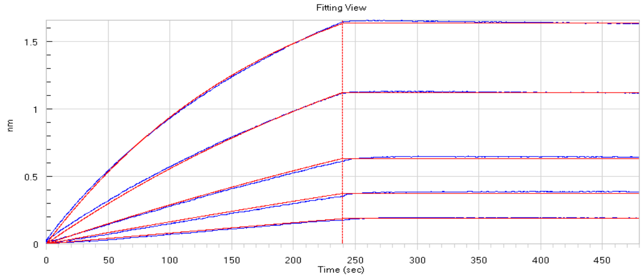

A/Victoria/361/2011 (H3N2) binding to H3v-47 IgG, K_d < 1 pM

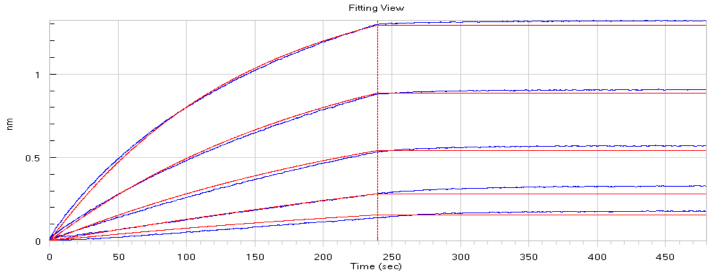

A/Perth/16/2009 (H3N2) binding to H3v-47 Fab, K_d < 1 pM

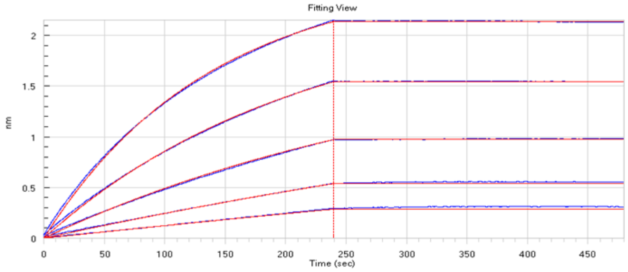

A/Perth/16/2009 (H3N2) binding to H3v-47 IgG, K_d < 1 pM

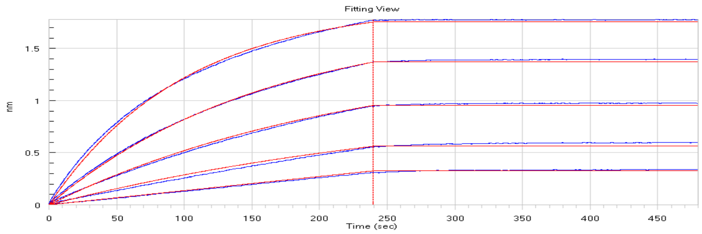

A/Hong Kong/1/1968 (H3N2) binding to H3v-47 IgG, K_d = 88.5 nM

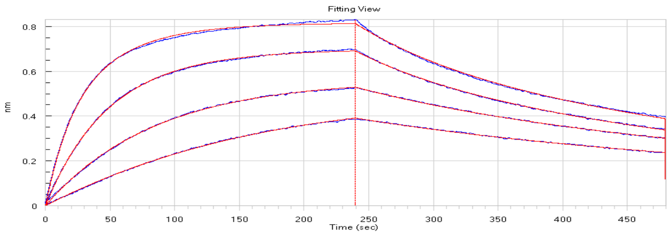

A/Victoria/3/1975 (H3N2) binding to H3v-47 IgG, K_d = 69.0 nM

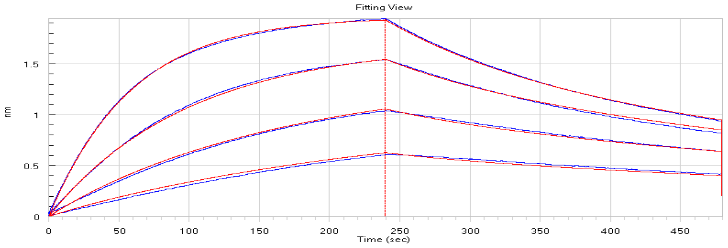

A/Bangkok/1/1979 (H3N2) binding to H3v-47 IgG, K_d= 85.0 nM

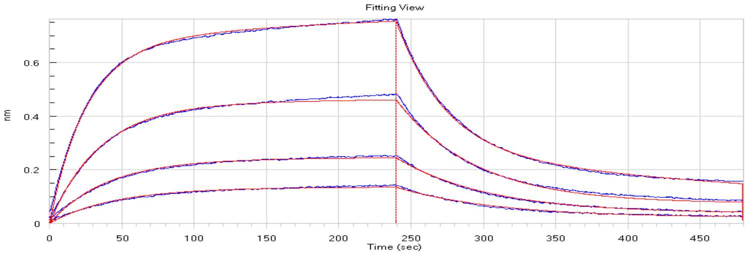

A/Leningrad/360/1986 (H3N2) binding to H3v-47 IgG, K_d = 130 nM

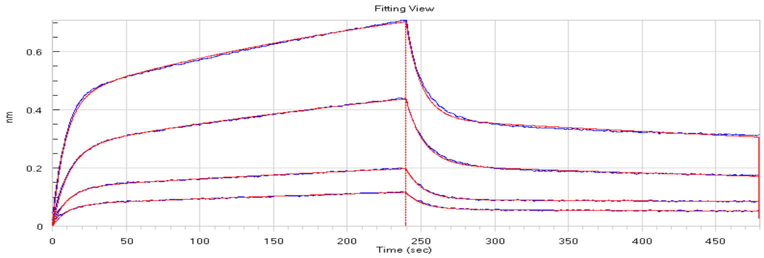

Binding to H3v-47 Fab was not detected (at 4,000 nM) for the following strain HAs:

A/Minnesota/11/2010 (H3N2) K82E mutant

A/Minnesota/11/2010 (H3N2) K82E/S124G mutant

A/Hong Kong/1/1968 (H3N2)

A/Victoria/3/1975 (H3N2)

A/Bangkok/1/1979 (H3N2)

A/Leningrad/360/1986 (H3N2)

A/Texas/36/1991 (H1N1)

A/Japan/305/1957 (H2N2)

A/Vietnam/1203/2004 (H5N1)

A/Shanghai/2/2013 (H7N9)

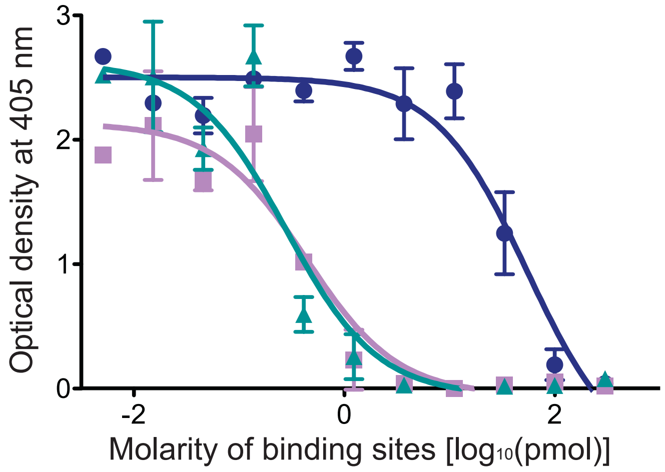

	H3v-47	IC_{50} (pmol)
●	Fab	54.4
■	$(Fab)_2$	0.44
▲	IgG	0.28

Supplementary Figure 2. Comparison of neutralizing activity exhibited by H3v-47 Fab, F(ab')$_2$ or IgG against A/Minnesota/11/2010 H3N2v virus. The amounts of H3v-47 Fab, F(ab′)$_2$ or IgG used for the neutralization assay were normalized for the number of binding sites present on each molecule, with a starting concentration of 15 μg/mL of the Fab, 16.5 μg/mL of F(ab′)$_2$ and 22.5 μg/mL of IgG. The antibody was diluted three-fold across the plate. The IC_{50} values were determined by non-linear regression analysis of log_{10} [inhibitor] vs. response function, using Prism software (GraphPad)

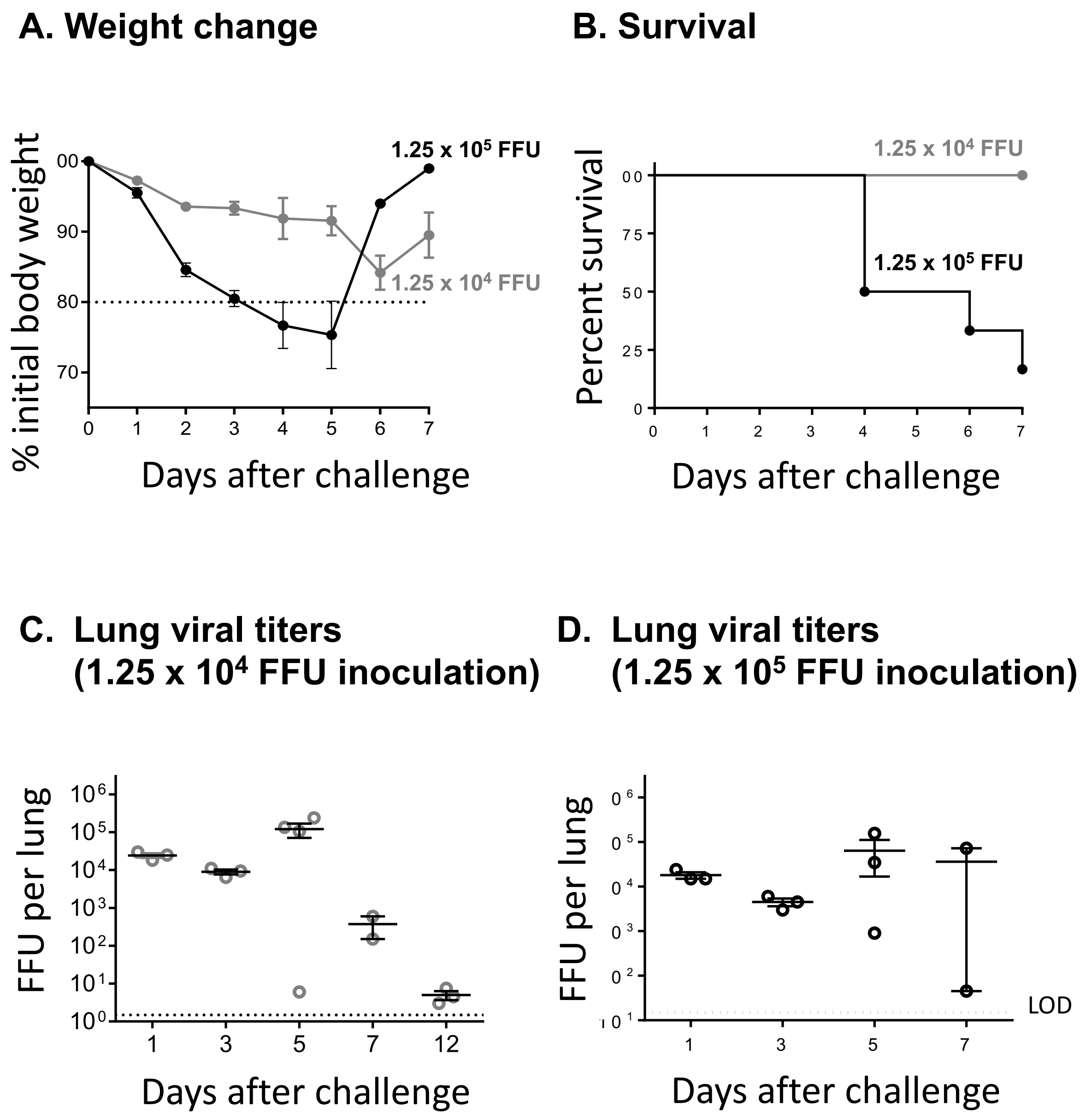

Supplementary Figure 3. Development of lethal respiratory challenge mouse model for H3N2v A/Minnesota/11/2010 virus. Two groups of female DBA/2J mice (n = 15 per group) were inoculated i.n. with ~ 1.2 x 10^5 (high dose), or ~1.2 x 10^4 (low dose) FFU of A/Minnesota/11/2010 X-203 virus stock and monitored for 7 days for weight change kinetics **(a)** and survival (**b**). The results showed a dose-dependent response to the virus, and the highest dose caused mortality. The figure shows body weight only for the animals that survived based on actual death between watches or endpoint for euthanasia. We also measured lung virus titers on dpi 1, 3, 5, 7, or 12 lungs (n = 3 per time point for each group) for the low dose (**C**) or high dose **(D).** The data showed the peak lung titers occurred on dpi 5, and mortality occurred during dpi 4-7.

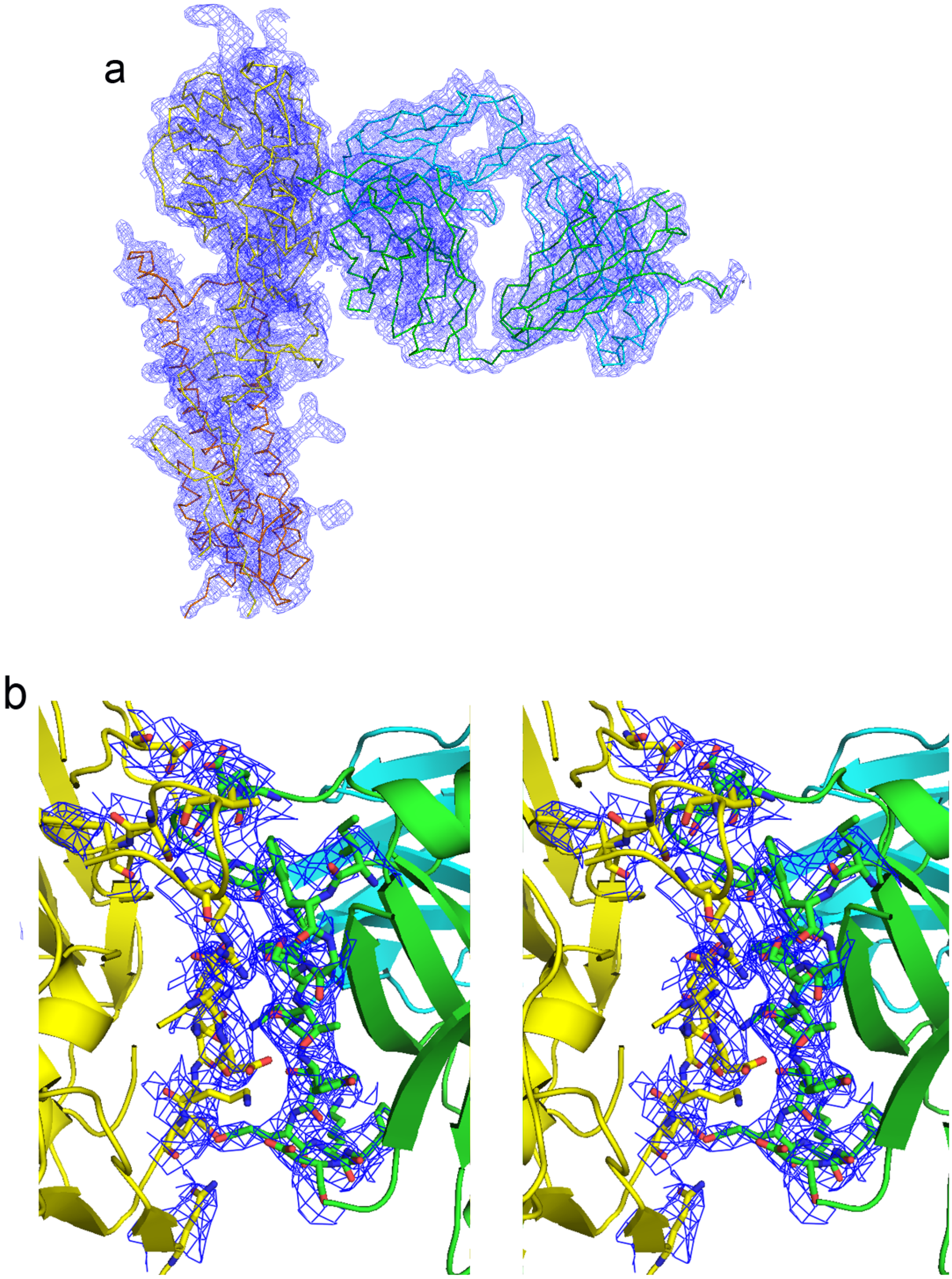

Supplementary Figure 4. Electron density map (2Fo-Fc) of H3v-47 Fab-H3N2v HA complex at 3.57 Å resolution contoured at a 1σ level. (**a**) Density map of overall structure of the complex. (**b**) Stereo image of the electron density map of the interacting residues (shown as sticks) in CDRs H2 and H3 and HA.

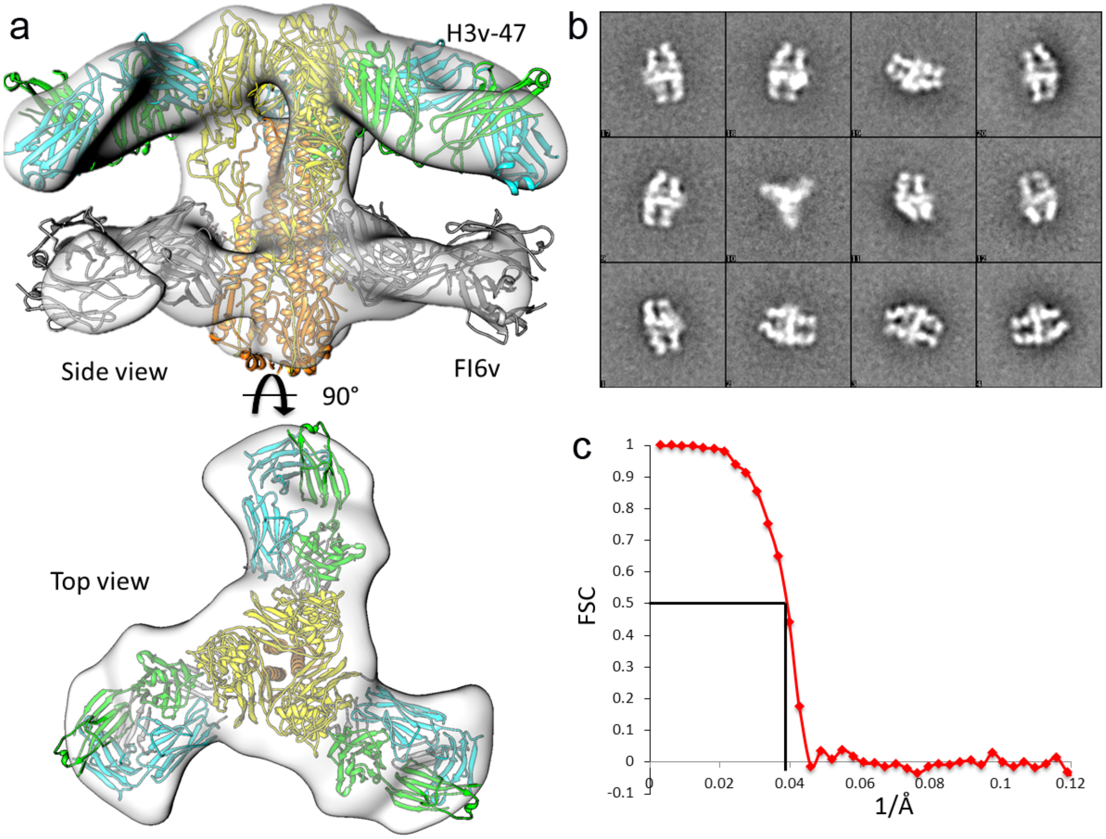

Supplementary Figure 5. Negative-stain EM reconstruction of H3v-47 Fab in complex with A/Minnesota/11/2010 H3N2v HA and FI6v Fab. (a) Reference-free 3D reconstruction of HA in complex with H3v-47 Fab and FI6v Fab. FI6v Fab that binds to HA stem is included in the EM reconstruction to provide additional mass and features and provide a comparison with the location of H3v-47 on the HA head domain. The crystal structure of the complex was fitted into the reconstruction with HA shown in orange (HA2) and yellow (HA1) and H3v-47 Fab heavy chain in green and light chain in cyan. FI6v Fab is shown in grey from its complex with H3N2 HA (PDB code 3ZTJ), with the HA removed. **(b)** Reference-free 2D class averages for A/Minnesota/11/2010 H3N2v HA in complex with Fabs H3v-47 and Fab FI6v. **(c)** Fourier shell correlation cutoff of 0.5 corresponds to a resolution of 25.8 Å.

a

H3v HA mutation	Binding of H3v-47	
	Fab (nM)	IgG (pM)
WT	44.3	< 1
K82E	NB	< 1
S124G	94.0	< 1
K82E/S124G	NB	< 1
Q122N	57.2	< 1
D133N	37.3	< 1
V144N	107.9	< 1
Q122N/D133N/V144N	227	< 1

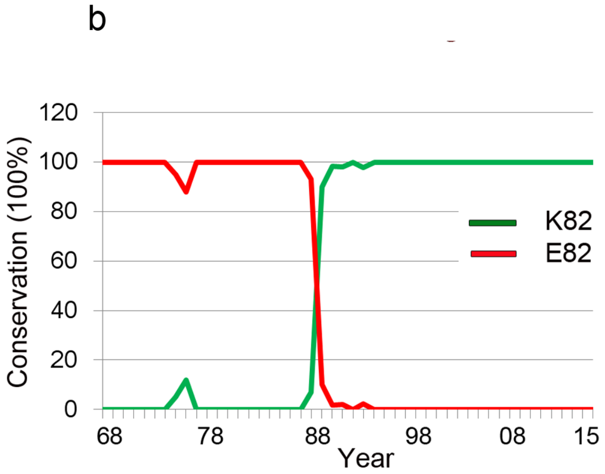

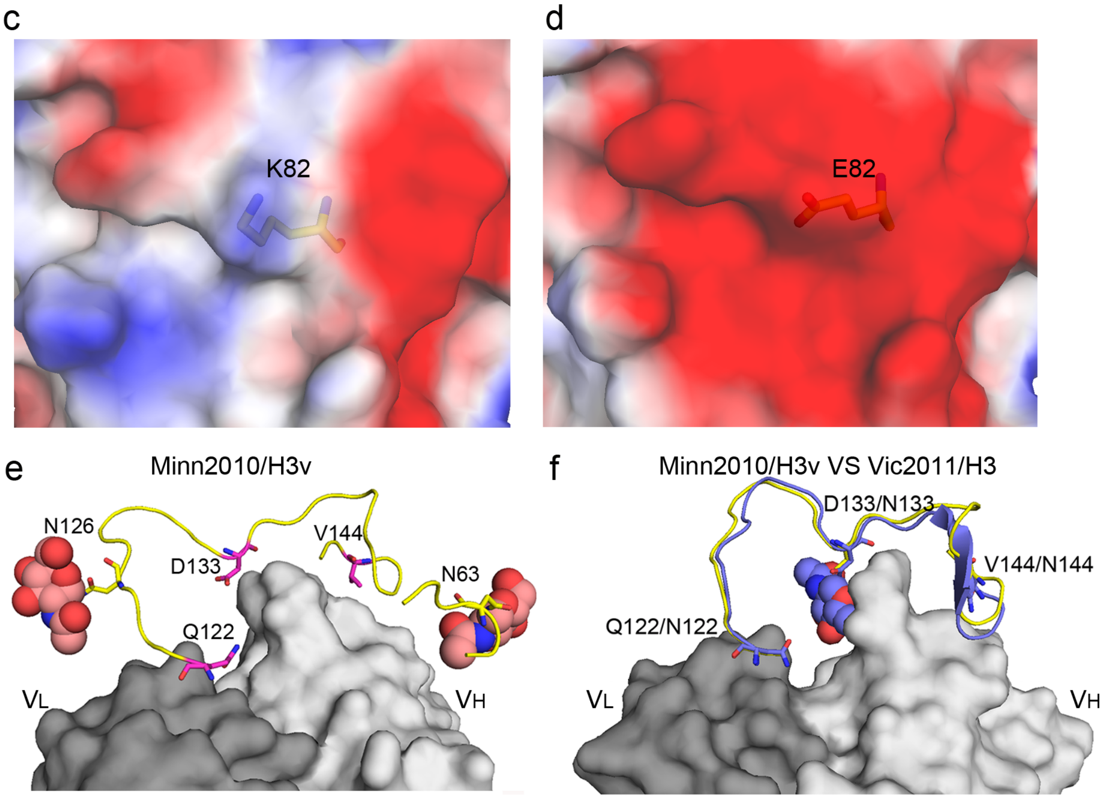

Supplementary Figure 6. Mutagenesis studies of the epitope residues of A/Minnesota/11/2010 H3N2v HA. (a) Affinity of H3v-47 binding to H3N2v HA wild-type (WT) or HA mutants, determined by biolayer interferometry. (b) Evolution of HA at position 82 in antigenic site E is driven by natural variation across human H3 HAs spanning 1968-2015. The switch from E82 to K82 occurred in 1988-1989. All full-length and non-redundant human H3N2 HA sequences were

obtained from the Influenza Virus Resource at the NCBI database for sequence conservation analysis. (c-d) Electrostatic potential surfaces around WT A/ Minnesota/11/2010 H3N2v HA with K82 (c) and A/Minnesota/11/2010 H3N2v HA with the K82E mutant model (d) (-6.1 to 6.1 K_bT/ec potential range). Electrostatic surface potentials were calculated using the APBS program[2]. Negatively charged regions are red; positively charged regions are blue. The K82E mutation alters the predicted electrostatic potential significantly from strong positive (c) to negative (d) around HA1 position 82, causing unfavorable predicted binding to H3v-47. (e) Close-up view of the only two N-glycosylation sites (N63 and N126) in the HA around the binding interface. A/ Minnesota/11/2010 H3N2v HA peptides are shown as cartoon in yellow, and H3v-47 Fab is shown as surface with heavy chain or light chain in light or dark gray, respectively. The two glycosylation sites N63 and N126 near the epitope do not make any contacts in the antibody-antigen interface. Residues Q122, D133 and V144 (highlighted in magenta) in the binding interface are not glycosylated in the HA of A/Minnesota/11/2010 H3N2v, but are glycosylated in most recent H3 strains (seen in Figure S6). (f) Structural superimposition of the A/ Minnesota/11/2010 H3N2v complex with A/Victoria/361/2011 HA (PDB code 4O5N, in blue) that also can be neutralized efficiently by H3v-47. Residues Q122, D133 and V144 in A/Minnesota/11/2010 H3N2v HA, correspond to the N-glycosylated N122, N133 and N144 residues in A/Victoria/361/2011 (only the glycan at N133 is modeled in the structure).

A

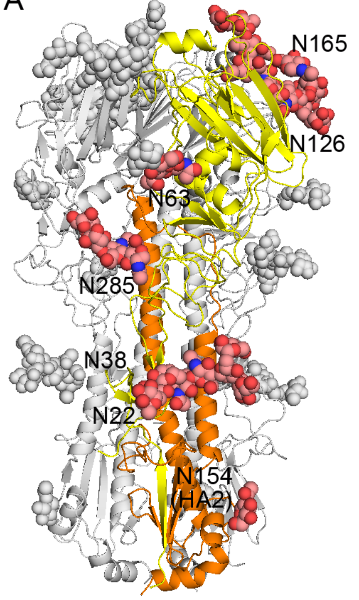

B

H3N2 strains	45	63	81	122	126	133	144	165	246
A/Minnesota/11/2010	S	✓		Q	✓	D	V	✓	✓
A/Hong Kong/1/1968			✓					✓	
A/Victoria/3/1975		✓			✓			✓	
A/Panama/2007/1999		✓		✓	✓	✓	✓	✓	✓
A/Wisconsin/67/2005		✓		✓	✓	✓	✓	✓	✓
A/Victoria/361/2011	✓	✓		✓	✓	✓	✓	✓	✓
A/Texas/50/2012	✓	✓		✓		✓	✓	✓	✓

Supplementary Figure 7. N-glycosylation sites in A/Minnesota/11/2010 H3N2v HA. (a) The predicted N-linked glycans (PNGs) on A/Minnesota/11/2010 H3N2v HA. The coloring of HA is essentially as in Figure 3a. Four potential N-glycosylation sites (HA1 N63, N126, N165 and N246) are in the head domain, and five in the stem [N8 (not shown in the structure), HA1 N22, N38, N285 and HA2 N154]. Glycans on the other protomers of the trimer are shown as white spheres. (b) Comparisons of Asn glycosylation in the globular head of A/Minnesota/11/2010 H3N2v HA with pandemic and representative seasonal H3 HAs. Only 4 potential N-glycosylation sites are present in the head domain of A/Minnesota/11/2010 H3N2v HA compared with 6-7 sites in recent seasonal H3 HAs. Q122, D133 and V144 in the paratope of H3v-47, which are glycosylation sites in other recent H3 HAs, are highlighted in red.

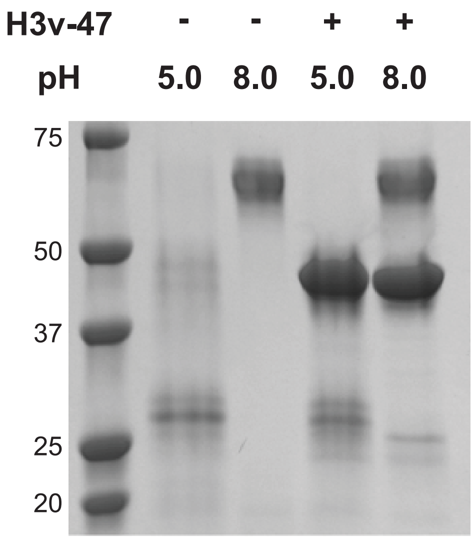

Supplementary Figure 8. H3v-47 does not block the low pH-induced conformational changes of the HA. Non-reducing SDS-PAGE of trypsin-digested A/Minnesota/11/2010 H3N2v HA in the absence (lanes 1 and 2) or presence (lanes 3 and 4) of H3v-47 Fab. The HA or HA-Fab mixture was exposed to pH 5.0 (lanes 1 and 3) or pH 8.0 (lanes 2 and 4) at 37°C for 1 h before trypsin digestion at pH 8.4. As expected, the HA was digested completely at pH 5.0 (lane 1), but at pH 8.0, the HA remained intact. After pre-complexing of the HA with H3v-47 Fab, the HA still was digested at pH 5.0 and stable at pH 8.0.

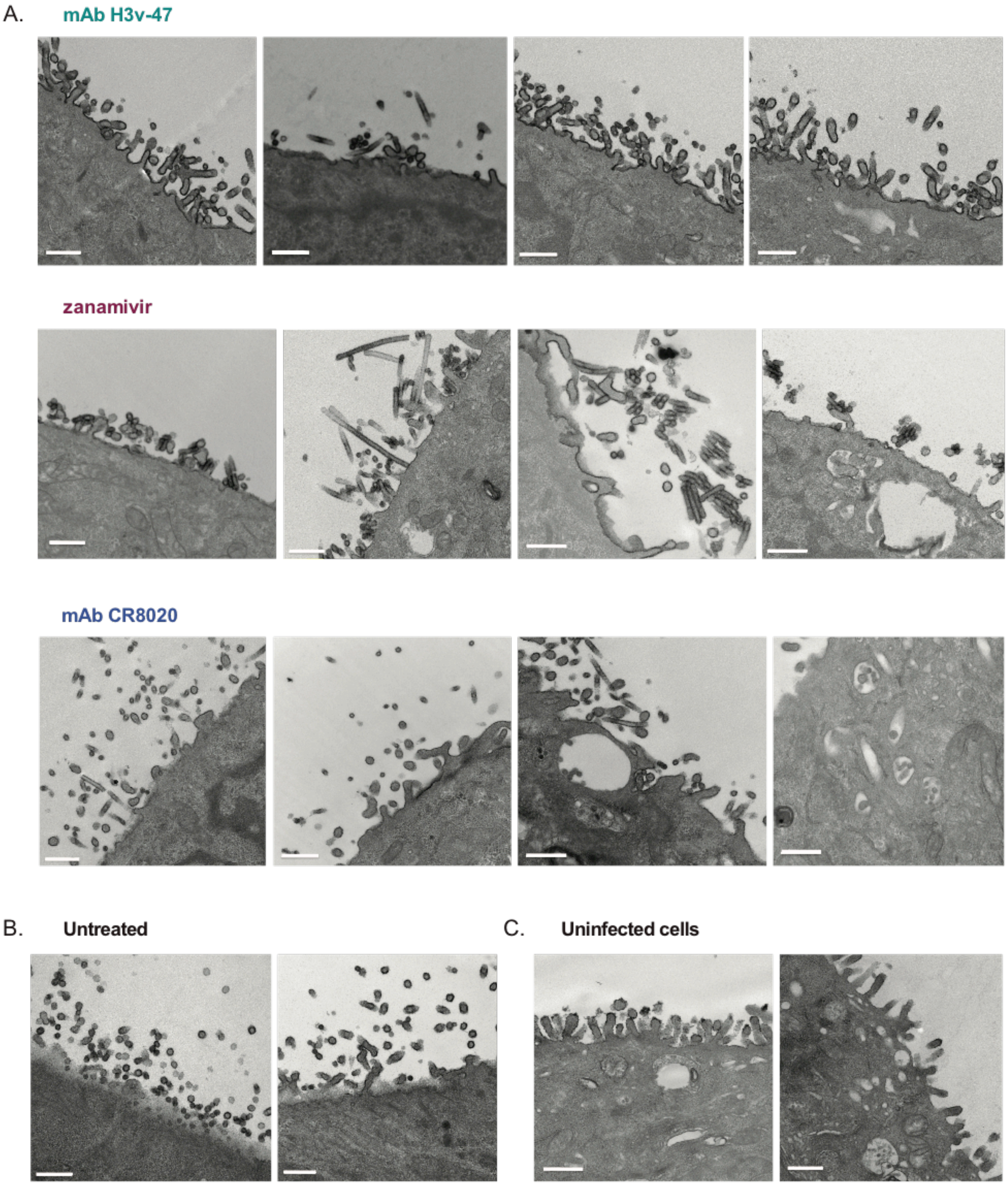

Supplementary Figure 9. TEM images of the surface of MDCK cells inoculated with A/ Minnesota/11/2010 H3N2v virus. H3N2v infected MDCK cells were incubated with **(a)** IgG of mAbs H3v-47 or CR8020 or by the small molecule inhibitor zanamivir or (b) plain Opti-MEM

at 3 h post-inoculation and fixed for imaging at 14 h. **(c)** Surface of uninfected MDCK cells treated similar to virus inoculated cells. Representative images of two independent experiments are shown. The white scale in each image represents 500 nm.

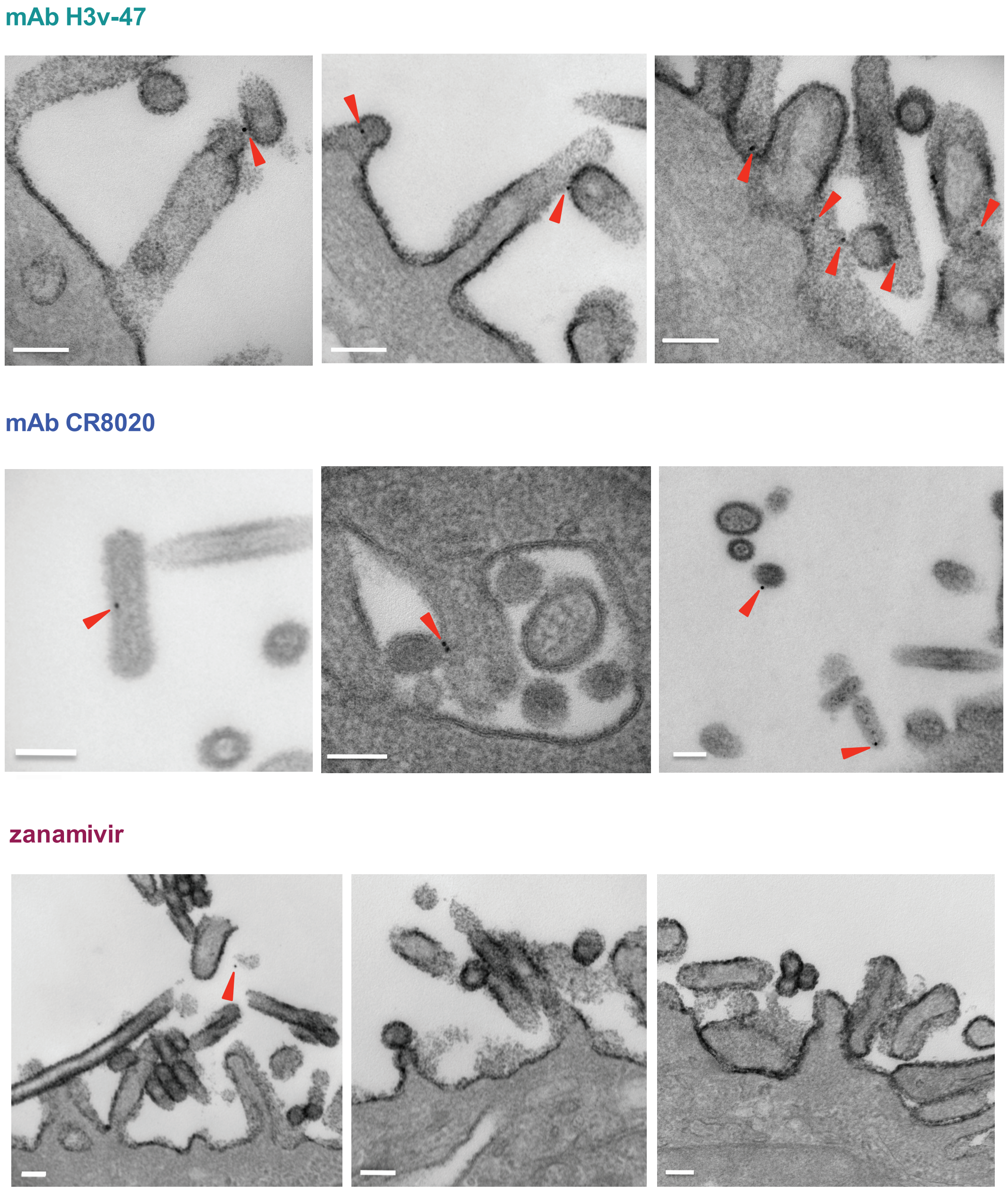

Supplementary Figure 10. TEM images of the surface of MDCK cells with gold labeling. TEM images are shown of the surface of MDCK cells that were inoculated with A/Minnesota/11/2010 H3N2v virus. The cells were incubated with IgG of mAbs H3v-47 or CR8020 or by the small molecule inhibitor zanamivir at 3 h post-inoculation. Anti-human IgG conjugated to 10 nm gold particles was added to the cells at 13 h post-inoculation and fixed after 1 h. The black opaque dots indicated by the red triangles represent the gold particles. The white scale in each image represents 100 nm.

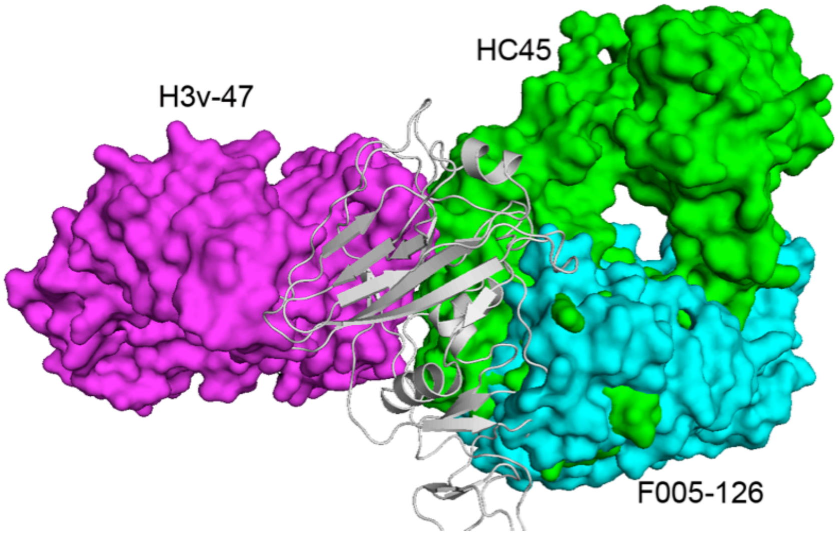

Supplementary Figure 11. Comparison of approach angle of H3v-47 (magenta) with two reported H3-neutralizing antibodies HC45 (green) and F005-126 (cyan) that bind to HA head domain but not to the RBS. The HAs from the complex of H3v-47 and A/Minnesota/11/2010 H3N2v HA, HC45-HA (PDB code 1QFU) and F005-126-HA (PDB code 3WHE) are aligned, and only A/Minnesota/11/2010 H3N2v HA is shown in grey cartoon for clarity. These antibodies (shown as surface) target the HA globular head with different approach angles.

References for Supplementary Information

1. Chen, V.B., Arendall, W.B., Headd, J.J., Keedy, D.A., Immormino, R.M., Kapral, G.J., Murray, L.W., Richardson, J.S. & Richardson, D.C. MolProbity: all-atom structure validation for macromolecular crystallography. Acta Crystallogr. D Biol. Crystallogr. 66, 12–21 (2010).

2. Baker, N.A., Sept, D., Joseph, S., Holst, M.J. & McCammon, J.A. Electrostatics of nanosystems: application to microtubules and the ribosome. Proc. Natl. Acad. Sci. U. S. A. 98, 10037–10041 (2001).
